# Connectivity and network state-dependent recruitment of long-range VIP-GABAergic neurons in the mouse hippocampus

**DOI:** 10.1038/s41467-018-07162-5

**Published:** 2018-11-28

**Authors:** Ruggiero Francavilla, Vincent Villette, Xiao Luo, Simon Chamberland, Einer Muñoz-Pino, Olivier Camiré, Kristina Wagner, Viktor Kis, Peter Somogyi, Lisa Topolnik

**Affiliations:** 1 Neuroscience Axis, CHU de Québec Research Center, Université Laval, Quebec, QC G1V 4G2 Canada; 2 Department of Biochemistry, Microbiology and Bio-informatics, Université Laval, Quebec, QC G1V 0A6 Canada; 3 Department of Pharmacology, Oxford University, Oxford, OX1 3QT UK

## Abstract

GABAergic interneurons in the hippocampus provide for local and long-distance coordination of neurons in functionally connected areas. Vasoactive intestinal peptide-expressing (VIP+) interneurons occupy a distinct niche in circuitry as many of them specialize in innervating GABAergic cells, thus providing network disinhibition. In the CA1 hippocampus, VIP+ interneuron-selective cells target local interneurons. Here, we discover a type of VIP+ neuron whose axon innervates CA1 and also projects to the subiculum (VIP-LRPs). VIP-LRPs show specific molecular properties and target interneurons within the CA1 area but both interneurons and pyramidal cells within subiculum. They are interconnected through gap junctions but demonstrate sparse spike coupling in vitro. In awake mice, VIP-LRPs decrease their activity during theta-run epochs and are more active during quiet wakefulness but not coupled to sharp-wave ripples. Together, the data provide evidence for VIP interneuron molecular diversity and functional specialization in controlling cell ensembles along the hippocampo-subicular axis.

## Abstract (web summary)

Long-range GABAergic hippocampal neurons project to cortical and subcortical areas, while vasoactive intestinal peptide (VIP+) interneurons can inhibit other GABAergic neurons. Here, authors discover a VIP+, long-range projecting GABAergic neuron whose activity is associated with quiet wakefulness.

## Introduction

Understanding brain computations during different cognitive states requires identifying cell types, their connectivity motifs and the recruitment patterns under different behavioural conditions. GABAergic inhibitory neurons play a pivotal role in cortical computations through gain control, sensory tuning and oscillatory binding of cell ensembles^1–4^. However, understanding cortical inhibition has been a challenging task as this process is executed through a diverse group of local and long-range projecting (LRP) GABAergic neurons^5^. Many types of GABAergic cells that have been identified by earlier investigations remain functionally uncharacterized. This is especially the case for sparse cell types, which represent a minority of the cortical neuronal population and, therefore, have not been frequently sampled in blind electrophysiological recordings. In particular, until recently, very little has been known about the functional organization of GABAergic cell types that are specialized in the selective coordination of inhibitory interneurons. These so-called interneuron-selective (IS) cells express vasoactive intestinal peptide (VIP) alone or in combination with calretinin^6,7^. They originate from the caudal ganglionic eminence and are the last cells to integrate into the cortical habitat^8,9^, where they innervate many different types of local interneurons, including the somatostatin (SOM+), calbindin (CB+), parvalbumin (PV+), VIP (VIP+) and calretinin (CR+) expressing GABAergic cells^6,7,10,11^. Development of novel transgenic and optogenetic technologies allowed to investigate how these cells can coordinate the operation of cortical microcircuits^12–17^. A common finding between different cortical regions is that VIP+ IS cells suppress some local interneuron activity during complex behaviours, including visual processing^12,14,16^, locomotion^13^ and reward-associated learning^17^, thus leading to network disinhibition. However, similar to other GABAergic cells, VIP+ neurons are diverse in properties^6,7,18–20^ and, likely, in circuit function. Yet, no attempt has been made for a detailed physiological and functional analysis of morphologically defined subtypes of VIP+ interneurons.

The hippocampal CA1 inhibitory circuitry can be considered one of the best characterized so far. Indeed, over the last three decades, the findings of multiple laboratories have culminated in a detailed wiring diagram of hippocampal CA1 GABAergic circuitry, with at least 21 inhibitory cell types identified to date^21^. Hippocampal CA1 VIP+ interneurons constitute two functionally different GABAergic cell populations: basket cells (BCs^22^) and IS interneurons (IS2 and IS3 cells^6^), which can modulate the activity of principal cells (PCs) or of different types of CA1 interneurons with a different degree of preference^23,24^. VIP+ BCs (VIP-BCs) can co-express cholecystokinin (CCK) and, in addition to targeting PC somata, can contact PV-positive BCs, indicating that VIP-BCs can exert both inhibitory and disinhibitory network influences^23^. In contrast, the VIP+ IS interneurons prefer to contact inhibitory interneurons^6^, and modulate interneuron firing properties^24^. Although disinhibition can be a common mechanism of hippocampal computations necessary for the induction of synaptic plasticity and memory trace formation and consolidation^25^, current findings indicate that its effect is mostly local due to the local innervation of hippocampal inhibitory microcircuits through VIP+ interneurons^24^. Interestingly, anatomical data point to the existence of long-range circuit elements that could account for cross-regional disinhibition between the hippocampus and functionally connected areas: CA1 SOM- or muscarinic receptor 2 (M2R)-expressing GABAergic cells innervate hippocampal inhibitory interneurons and can project to several cortical and sub-cortical areas, including the rhinal and retrosplenial cortices, subiculum (SUB) and medial septum (MS)^26–30^. Despite the considerable recent interest in LRP GABAergic neurons, very little is currently known about the connectivity and function of these cells during different network states in awake animals.

Here, we reveal a subtype of VIP-expressing LRP (VIP-LRP) GABAergic neuron that exhibits a specific molecular profile and innervates, in addition to the hippocampal CA1, the SUB, with region-specific target preference. Functionally, VIP-LRP cells correspond to theta-off cells^31,32^ as they decrease their activity during theta-run epochs associated with locomotion and exhibit high activity during quiet wakefulness. The identification of this circuit element reveals an additional mechanism for the behaviour- and network-state-dependent inter-regional coordination of activity within the hippocampal formation.

## Results

### VIP-LRP neuron in the CA1 hippocampus

**Fig. 1:**
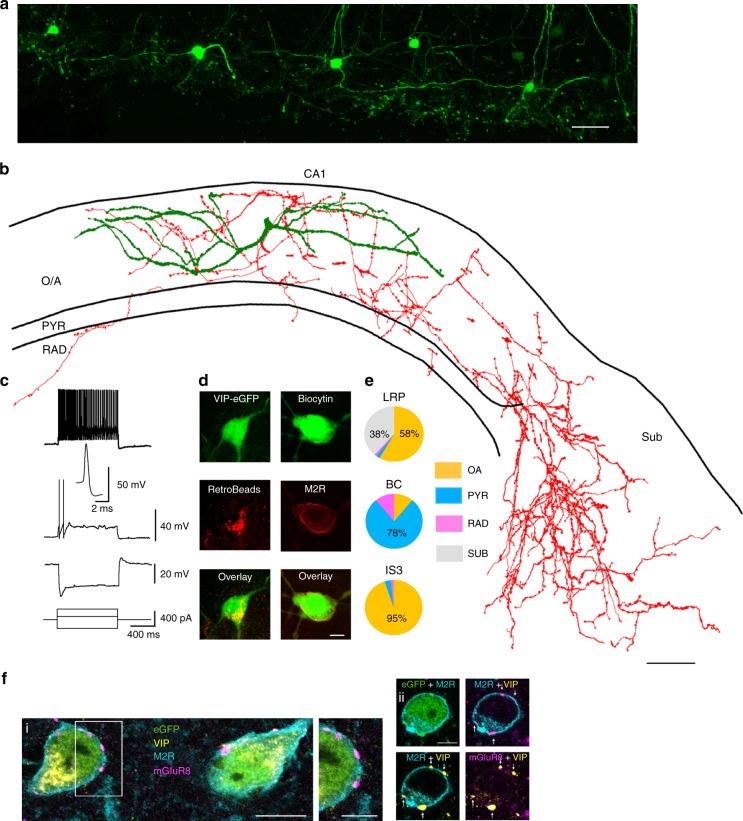
Identification of VIP-LRPs in the VIP-eGFP mouse. **a** Two-photon image (maximal projection of a z-stack of 200 µm height) of the CA1 area from an acute hippocampal slice (300 µm) of a VIP-eGFP mouse showing the location of GFP cell bodies, axons and dendrites in the O/A area of CA1. Scale bar: 100 µm. **b** Reconstruction (the axon is shown in red, the dendrites are shown in green) of a VIP-LRP cell that was recorded and filled with biocytin in a slice obtained from a VIP-eGFP mouse. Scale bar: 100 µm. **c** Representative voltage responses of a VIP-LRP to hyperpolarizing (−240 pA), and depolarizing (+80 pA and +280 pA) current injections, with an inset illustrating the first spike evoked by +80-pA current pulse. **d** Confocal images showing RetroBeads labelling of a VIP-LRP soma (left) after injection in subiculum and immunoreactivity for M2R in a VIP-positive neuron labelled with biocytin (single focal plane, right). Scale bar: 10 µm. **e** Pie charts illustrating the mean axonal distribution in different layers (based on axon length obtained following reconstruction in Neurolucida) for groups of cells corresponding to three different cell types: VIP-LRP (*n* = 10), VIP-BC (*n* = 5) and IS3 cell (*n* = 6). OA oriens-alveus, PYR stratum pyramidale, RAD stratum radiatum, SUB subiculum. No axon was detected within stratum lacunosum moleculare (LM) for the three cell types. Statistically significant differences in the axon distribution between VIP-LRP and VIP-BCs, VIP-LRP and IS3, and VIP-BCs and IS3 at **p < 0.01, one-way ANOVA followed by Tukey’s test. **f** VIP-LRP cells, identified by somato-dendritic membrane M2R immunoreactivity (blue), are innervated by terminals rich in presynaptic mGluR8 (purple). Single optical slices (0.45 mm thick) of confocal images of quadruple immunoreactions as indicated: i, right, framed area at higher magnification; ii, four VIP+ terminals (arrows) show mGluR8 immunoreactivity. Scale bars: 10 µm (left), 5 µm (middle), 5 µm (right)

To characterize the electrophysiological and morphological properties of VIP+ interneurons in the hippocampal CA1 area, we first performed patch-clamp recordings from VIP+ cells in acute slices obtained from VIP-eGFP mice (Fig. 1a; Supplementary Figs. 1; 2g, i; the expression of GFP in this mouse strain was characterized previously^24^). Following biocytin labelling, 97 VIP-GFP+ interneurons were visualized and identified as BCs, IS3 cells or as novel LRP neurons (VIP-LRP; Fig. 1b; Supplementary Fig. 1b; Supplementary Table 1). The VIP-LRP neurons typically occurred at the oriens/alveus (O/A) border and had horizontal sparsely spiny dendrites, which were mostly restricted to the stratum oriens (Fig. 1b). Their axon formed a local arbor in O/A and extended slightly into strata pyramidale (PYR) and RAD of the CA1 region. The main axon was partially myelinated and travelled outside the CA1, giving rise to a large axon cloud in the proximal SUB (Fig. 1b; *n* = 40 cells out of 78 biocytin-filled O/A VIP-GFP+ interneurons). Thus, in contrast to VIP-BCs and to IS3 cells, the axon of VIP-LRP neurons occupied two major areas: CA1 O/A and SUB (Fig. 1b, e). The total length of the axonal arbor in a 300-µm slice was between 7704 and 36,167 µm (no shrinkage correction). The axon length occupying the CA1 vs SUB was 2000–20,000 (median ± SD: 10,184 ± 5002) and 1000–26,000 (median ± SD: 5041 ± 6740) µm, respectively; the large variability was likely due to a different degree of the axon preservation in slices (Fig. 1e; *n* = 10 cells). These cells showed a regularly spiking firing pattern and a membrane potential ‘sag’ in response to a hyperpolarizing step to −100 mV (Fig. 1c; Supplementary Fig. 1a). Furthermore, their intrinsic membrane properties were similar to those of VIP-BCs [except for the sag and the fast afterhyperpolarization (AHP) amplitude] but differed in many parameters from IS3 cells (Supplementary Table 1). To provide additional evidence for the presence of SUB-projecting VIP+ neurons in the CA1 O/A, we injected red RetroBeads in the SUB of VIP-eGFP mice. In addition to pyramidal cells, bead-labelled VIP-GFP-positive neurons were detected in the CA1 O/A area (Fig. 1d, left), thus confirming that a population of O/A VIP+ neurons sends long-range axons to the SUB. As to their molecular profile, all VIP-LRP cells tested with an axon reaching SUB were immunopositive for muscarinic receptor 2 (M2R; *n* = 7/7 cells; Fig. 1d, right), thus identifying the M2R as an additional molecular marker of VIP-LRPs. Furthermore, a large fraction of M2R+/VIP-GFP+ cells co-expressed CB (29/53 cells; Supplementary Fig. 2a, i) but were negative for CCK (Supplementary Fig. 2d, i), nitric oxide synthase (NOS; Supplementary Fig. 2e, i), CR (Supplementary Fig. 2f) and SOM (Supplementary Fig. 2h, i). This was in contrast to VIP-BCs and IS3 cells, which co-expressed CCK or CR, respectively, and were negative for M2R and CB (Supplementary Fig. 2b, c). Overall, ~50% of VIP-GFP+ cells in the CA1 O/A of VIP-eGFP mouse were co-expressing M2R (Supplementary Fig. 2i), corresponding to the VIP-LRP population. In the rat, trilaminar cells projecting to the subiculum are rich in M2R in the somato-dendritic membrane and are innervated by presynaptic mGluR8-positive terminals^33^. We tested if the VIP-GFP-M2R-positive cells in the mouse received mGluR8+ input and found that most VIP-GFP-M2R-positive cells in O/A were decorated by mGluR8+ terminals, some of which were themselves VIP-positive as in the rat (Fig. 1f). Some M2R+ neurons in O/A were not immunoreactive for VIP and GFP, and not all VIP-GFP+ neurons showed M2R immunoreactivity (Supplementary Fig. 2i), pointing to additional molecular diversity within M2R+ and VIP+ neuronal populations.

### Local connectivity of VIP-LRP cells

**Fig. 2:**
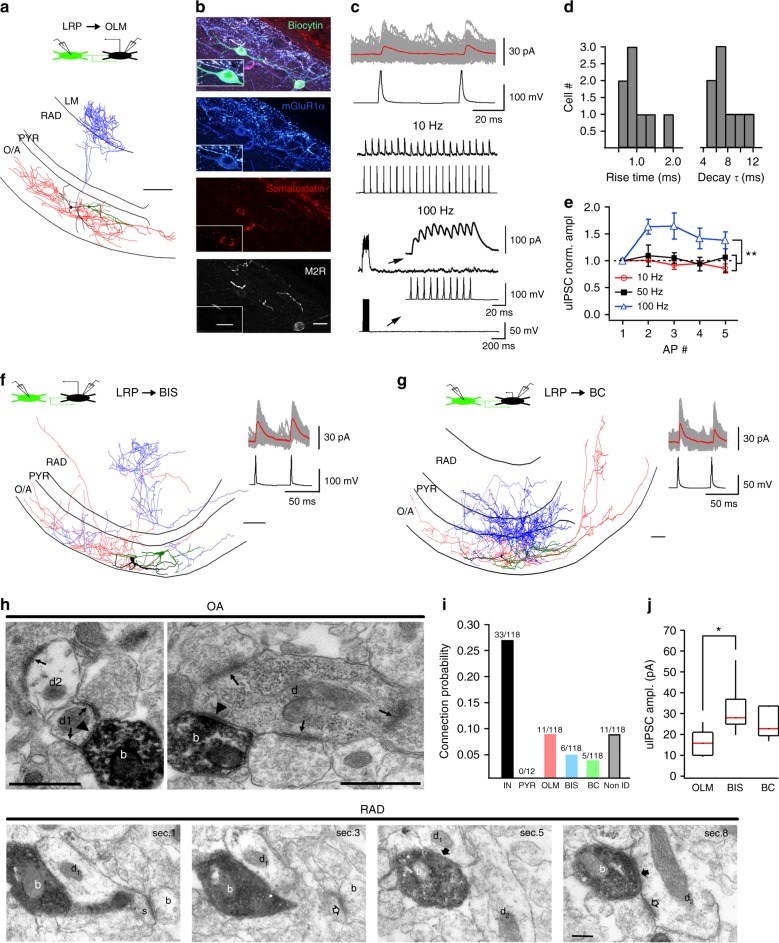
VIP-LRPs provide inhibition to CA1 O/A interneurons. **a** Reconstruction of a connected pair of VIP-LRP and O-LM interneurons. The axon of the VIP-LRP cell is shown in red and its dendrites are shown in green. The axon of the O-LM cell is shown in blue, with its dendrites shown in black. Scale bar: 100 µm. The schematics on top illustrates the configuration of recording. **b** Post hoc immunohistochemical analysis of a connected pair of VIP-LRP (immunoreactive for M2R) and O-LM cell, with insets showing the O-LM cell expanded. Scale bars: 20 µm, 10 µm (inset). **c** Representative traces of uIPSCs in an O-LM cell (100 consecutive traces with the average shown in red) evoked by two APs in a presynaptic VIP-LRP cell (top), and examples of uIPSCs during different frequencies of VIP-LRP firing: 10 Hz (middle) and 100 Hz (bottom). Insets at the bottom show expanded uIPSCs during 100-Hz firing of VIP-LRP. **d** Cumulative histograms of uIPSC rise time and decay time constant in O-LM cells (*n* = 11 pairs). **e** Summary plot (mean ± SEM) showing changes in uIPSC amplitude in O-LM cells during different firing frequencies of VIP-LRPs (**p < 0.01, one-way ANOVA/Tukey’s test). **f**, **g** Reconstructions of connected pairs of VIP-LRP and a bistratified (BIS; **f**) or basket (BC; **g**) cells. Scale bars: 100 µm. The insets on the right show corresponding uIPSCs. **h** Electron micrograph (EM) images of biocytin labelled boutons (**b**) of VIP-LRP cells. Top, the VIP-LRP boutons, which form type-2 synapses (arrowheads) with a small (left d1) and a large diameter (right) dendritic shafts (**d**) of interneurons receiving type-1 synapses (arrows) in stratum oriens. Bottom, EM images illustrating dendrites (d1, d2) in CA1 RAD as postsynaptic targets of a VIP-LRP. The VIP-LRP bouton (white ‘b’) makes two type-2 synapses (solid arrows): with a spiny (d1, s) and aspiny dendrite (d2) receiving a type-1 synapse (open arrow). Scale bars: 0.5 µm (top), 200 nm (bottom). **i** Summary graph illustrating the connection probability. **j** Boxplots representing the uIPSC amplitude for different targets (OLM: *n* = 8, BIS: *n* = 5, BC: *n* = 4; *p < 0.05, unpaired *t* test)

To determine the VIP-LRP physiological function, we next examined its local connectivity using simultaneous paired recordings and electron microscopic (EM) analysis (Fig. 2). Dual whole-cell patch clamp recordings showed that out of 118 attempts, 33 pairs of VIP-LRPs and CA1 O/A interneurons were connected synaptically, and no connection was found with CA1 PCs (Fig. 2i). Among the VIP-LRP targets, we identified different types of dendritic inhibitory cells (DT-INs), such as O-LM (Fig. 2a–e) and bistratified cells (BIS; Fig. 2f), and the perisomatic terminating interneurons (ST-INs), such as BCs (Fig. 2g). As O-LM cells were the most frequent target, we characterized the VIP-LRP to O-LM cell connection in more detail (Fig. 2a–e). The VIP-LRP synapses occurred on dendritic shafts of OLMs (distance from soma: 63.4 ± 19.8 µm, *n* = 7 pairs; Fig. 2a), had mean unitary inhibitory postsynaptic current (uIPSC) amplitude of 16.3 ± 2.4 pA (0 mV holding potential) and a failure rate of 60.1 ± 4.1% (*n* = 8 pairs). uIPSCs had slow kinetics consistent with the dendritic location of synapses (Fig. 2d). During repetitive VIP-LRP firing, uIPSC showed no change at 10–50 Hz but summated efficiently at 100 Hz (Fig. 2c, e). Apart from the uIPSC amplitude, which was higher in BISs (32.1 ± 6.2 pA, *n* = 5 pairs; Fig. 2j), the properties of VIP-LRP synapses were similar among different postsynaptic targets (Supplementary Table 3), and, also, did not differ significantly from synapses made by IS3 cells on O/A interneurons^24^.

To further validate the results of paired recordings, EM analysis of 39 synaptic junctions within the CA1 area made by two VIP-LRPs filled with biocytin was performed. The data showed that interneuron dendrites were frequent synaptic targets of VIP-LRPs. Of the 18 postsynaptic dendrites tested from the targets of one VIP-LRP, 16 originated from interneurons and 2 were unidentified (Fig. 2h, top). The other VIP-LRP cell made synapses with 6 interneuron dendrites and 15 unidentified dendrites, some of which emitted spines (Fig. 2h, bottom), suggesting that spiny interneurons or PCs could be among the VIP-LRP targets. Taken together, these data indicate that, locally, VIP-LRPs prefer to target interneurons and constitute a novel type of IS cells in the hippocampus.

**Fig. 3:**
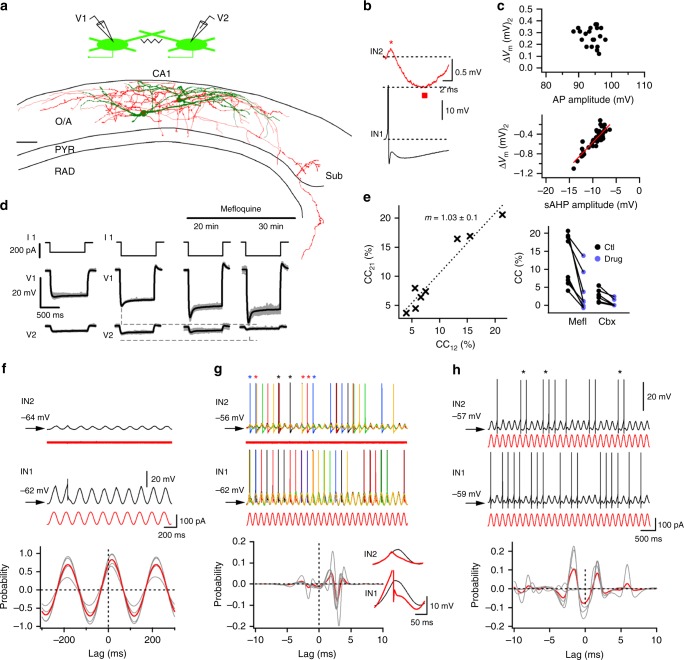
Electrical coupling between VIP-LRPs. **a** Schematic of the simultaneous recording of two VIP-LRPs and their reconstruction. Scale bar: 100 µm. **b** Representative example of the simultaneous recording of two VIP-LRPs with an AP initiated in one cell (IN1, black) and a corresponding voltage response in the second cell (IN2, red). The positive and negative components of the spikelet are shown with a star and square symbols, respectively. **c** Summary plots (*n* = 17 cells) indicating changes in the postsynaptic *V*_m_ as a function of the AP amplitude (upper) or sAHP amplitude (bottom). Red line is a linear fit to the data points (*r* = 0.88, Pearson correlation) for a spikelet component associated with sAHP in the presynaptic cell. **d** Representative examples of voltage traces recorded in the VIP-LRP pair before and after the application of mefloquine. **e** Summary plots for the coupling coefficient (CC) (left; *m*, slope of the regression line ± SE), and for the gap-junction blockers’ effect [right; Mefloquine (Mefl), *n* = 8; Carbenoxolone (Cbx) *n* = 6; *p* < 0.05; paired *t*-test]. **f** Voltage responses recorded in a VIP-LRP pair to a sinusoidal current (red trace, 5 Hz) applied to the IN1. The plot below shows cross-correlations in *V*_m_ between the two cells (*n* = 6 pairs), with red trace showing the average data. **g** Voltage responses (five consecutive traces of different colours superimposed) recorded in the VIP-LRP pair with the postsynaptic cell (IN2) being depolarized, and a sinusoidal current (red trace) applied to the IN1. Stars of different colours above the IN2 traces indicate APs generated synchronously in two cells. The plot below shows cross-correlations in the AP occurrence (*n* = 6 pairs), with red trace illustrating the average data. Insets on the right show voltage responses in two cells with (red trace) and without (black trace) an AP generated in the presynaptic cell. **h** Voltage responses recorded in a VIP-LRP pair to a sinusoidal current (red trace) applied to both cells. Stars above the IN2 trace indicate APs generated synchronously. The plot below shows cross-correlations in the AP occurrence (*n* = 6 pairs), with red trace showing the average data

To investigate the possible connectivity within the VIP-LRP population, we performed dual whole-cell patch-clamp recordings from VIP-GFP+ pairs (Fig. 3a). Our data showed that out of 36 attempts, 16 VIP-LRP pairs were connected through symmetric gap junctions with a coupling coefficient of 0.11 ± 0.1 (Fig. 3d, e), and one pair was connected synaptically. Electrotonic coupling between VIP-LRPs was blocked by the gap-junction inhibitor mefloquine^34^ (to 21.05 ± 11.5% of control, *n* = 8 pairs; *p* < 0.01; paired *t* test; Fig. 3d, e) or the broad-spectrum gap-junction blocker carbenoxolone (to 14.9 ± 10.3% of control, *n* = 6 pairs; *p* < 0.01; paired *t* test; Fig. 3e). Furthermore, the electrotonic signal conduction exhibited low-pass filter properties. As such, fast action potentials (APs) generated in cell 1 were strongly attenuated in cell 2, whereas the slow AHPs were better conducted, leading to a substantial hyperpolarization of cell 2 (Fig. 3b, c, g, bottom right insets). We then examined how a sinusoidal excitatory input modulated at theta-like frequency can be integrated by the electrically coupled VIP-LRP neurons (Fig. 3f–h). We found that, when both cells were kept at rest, a sinusoidal input applied to cell 1 induced subthreshold synchronous fluctuations of the membrane potential of cell 2, but was not able to drive its firing (Fig. 3f). When cell 2 was slightly depolarized to allow for spontaneous firing, the two VIP-LRPs could occasionally fire together but their coupling remained weak, and most spikes occurred asynchronously due to the AHP-associated inhibition of cell 2 (Fig. 3g). The voltage fluctuation in cell 2 was significantly higher when spikes were not generated in cell 1 [voltage peak without AP in cell 1: 8.5 ± 0.1 mV (inset black trace) vs voltage peak with AP in cell 1: 6.9 ± 0.1 mV (inset red trace), *n* = 6 pairs; *p* < 0.01; paired *t* test; Fig. 3g, bottom right inset]. When both cells received a synchronous theta-modulated excitatory input, their firing increased (to 112%, *n* = 6 pairs; Fig. 3h), but the spike synchrony remained weak (Fig. 3h, bottom). Together, these data indicate that electrotonic coupling between VIP-LRPs is unlikely to synchronize their recruitment in response to the theta-like input. Whether this may be the case at a different firing frequency^35,36^, activity-dependent state of the gap junctions or network size will need to be explored using computational modelling^37^.

### Distant connectivity of VIP-LRP cells

**Fig. 4:**
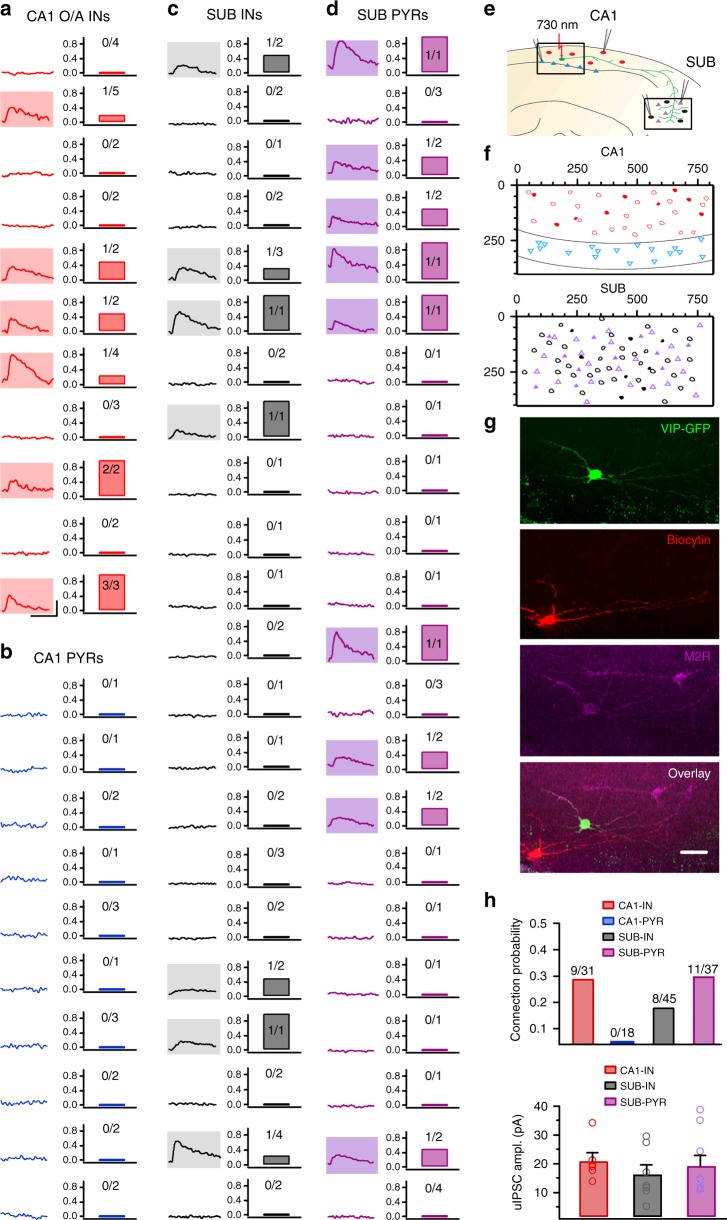
Two-photon glutamate uncaging-based mapping of local and distant axonal targets of VIP-LRPs. **a**–**d** Average traces of glu-IPSCs (*V*_hold_: 0 mV) evoked by uncaging of MNI-Glu on VIP+ O/A interneuron somata (left) and the corresponding connection probability (right) in CA1 O/A interneurons (**a**), CA1 PCs (**b**), SUB interneurons (**c**) and SUB PCs (**d**). Each row corresponds to a single cell with the ratio of connections indicated at bar graphs. Each recorded cell was tested for receiving input from 1 to 5 VIP-GFP+ O/A interneurons. Traces with shadow area correspond to examples of glu-IPSCs: CA1 interneurons (*n* = 9 connections out of 31 tested/11 cells), CA1 PCs (*n* = 0 connections out of 18 tested/10 cells), SUB interneurons (*n* = 8 connections out of 45 tested/26 cells), SUB PCs (*n* = 11 connections out of 37 tested/24 cells). Scale bars (shown in **a**): 20 pA, 10 ms. **e** Schematic of simultaneous patch-clamp recordings from different CA1 and SUB targets and two-photon MNI-Glu uncaging on somata of VIP-GFP+ cells in CA1 O/A. **f** Summary spatial maps illustrating the density of connections within the CA1 (top) and SUB (bottom). Connected cells are shown as shaded symbols. Scales are in µm. **g** Post hoc immunohistochemical validation of connected interneurons confirmed that VIP-GFP+ cells innervating CA1 O/A interneurons were positive for M2R and, thus corresponded to VIP-LRPs. Scale bar: 20 µm. **h** Summary bar graphs showing the connection ratio (top) and the peak amplitude (mean ± SEM) of glu-IPSCs (bottom) for different postsynaptic targets in CA1 and subiculum (CA1-IN *n* = 6, SUB-IN *n* = 7, SUB-PYR *n* = 9; *p* = 0.356, one-way ANOVA followed by Kruskal–Wallis test). The connection ratio is a ratio between the number of connections over the total number of tests for a given target type

To determine the distant targets of VIP-LRPs in SUB, we conducted single-cell two-photon glutamate uncaging-based mapping of connections by combining the photoactivation of CA1 O/A VIP-GFP+ cells and patch-clamp recordings of interneuron and PC targets (Fig. 4). As reported previously^38^, two-photon uncaging (730 nm, 20–30 mW/180 ms laser pulses) of locally delivered MNI-Glu [micropressure pulses (5 psi, 5 ms) via a glass pipette with tip diameter of 2–3 μm positioned ∼10 μm above the cell of interest] triggered single spikes in VIP-GFP+ interneurons, resulting in fast uncaging-evoked IPSCs (glu-IPSCs; delay onset: 4.8 ± 1.8 ms) in target cells in case of connection (Fig. 4a–e). Depending on the number of VIP-GFP+ cells per slice, this approach allowed us the testing of several VIP-GFP+ connections to a given target (1–5; Fig. 4a–e). First, consistent with our findings using paired recordings and ultrastructural analysis (Fig. 2), in the CA1 area, VIP-LRPs were connected to O/A interneurons (*n* = 9 connections out of 31 tested/11 cells; Fig. 4a, f). Glu-IPSCs were not detected in CA1 PCs (*n* = 0 connections out of 18 tested/10 cells; Fig. 4b, f), confirming the interneuron preference of VIP-LRPs. In addition, post hoc immunohistochemical examination of connected neurons revealed that VIP-GFP+ cells that were connected to O/A interneurons were positive for M2R (Fig. 4g), consistent with the neurochemical profile of VIP-LRPs (Figs. 1d and 2b). Surprisingly however, in the SUB, VIP-LRPs innervated both interneurons (*n* = 8 connections out of 45 tested/26 cells; Fig. 4c, f) and PCs (*n* = 11 connections out of 37 tested/24 cells; Fig. 4d, f) identified based on their dendritic and axonal properties. These data highlight the region-specific target preference of VIP-LRPs (Fig. 4h, top). The glu-IPSC amplitude was similar between all CA1 and SUB targets (Fig. 4h, bottom), although some target-specific differences were observed within a population of CA1 interneurons (Fig. 2j; Supplementary Table 3).

### Activity of VIP-LRP cells in awake mice

**Fig. 5:**
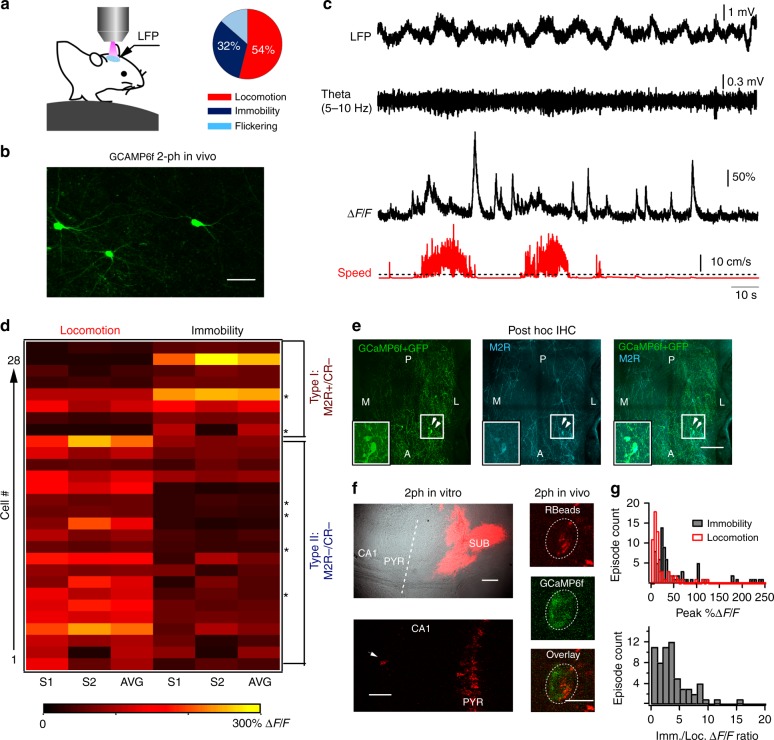
Imaging VIP-LRP activity in awake mice. **a** Schematic of two-photon Ca^2+^-imaging and LFP recordings in awake mice (left) and a pie-chart illustrating the time distribution of animal activity (right). **b** Two-photon image of the GCaMP6f-expressing VIP cells in CA1 O/A (maximal projection of a 100-µm Z-stack). Scale bar: 50 µm. **c** Representative traces of simultaneous LFP recording (upper raw trace and filtered for theta: 5–10 Hz), somatic Ca^2+^-imaging from a VIP cell, and animal speed (red). **d** VIP O/A cell segregation based on the peak Ca^2+^-transient during locomotion and immobility. Two types of neurons were revealed: type I VIP cells (*n* = 8) were positive for M2R and negative for CR (2 out of 2 cells tested indicated with stars) whereas type II VIP-expressing cells (*n* = 20) were negative for both M2R and CR (4 out of 4 cells tested). S1 and S2 represent two imaging sessions and AVG is the average peak Ca^2+^-signal during two sessions. **e** Confocal stitching of the CA1 O/A imaging window used for two-photon image acquisition in vivo and processed for post hoc immunohistochemistry illustrating GFP (left), M2R (middle) and the overlay of the two (right). White arrowheads point to M2R-positive VIP cells targeted with GCaMP6f (expanded in insets). Anatomical landmarks are indicated as follows: A—anterior, P—posterior, M—medial, L—lateral. Scale bar: 100 µm. **f** Representative two-photon images obtained from hippocampal slices of *VIP-Cre* mice in vitro (left) and from the CA1 O/A VIP+ interneuron in vivo (right) illustrating the RetroBead injection site in SUB (left top, Dodt image superimposed with epifluorescence image for Retrobeads), the retrogradely labelled CA1 PCs and an O/A interneuron (white arrow; left bottom) and a CA1 O/A VIP-LRP labelled with RetroBeads and expressing GCaMP6f (right). Scale bars: 500 µm (top), 50 µm (bottom left), 10 µm (bottom right). **g** Histograms of Ca^2+^-transient peak amplitude (top) and its ratio during immobility to that during locomotion (bottom) obtained from VIP-LRPs labelled with RetroBeads (*n* = 5 cells, 25–30 locomotion/immobility periods/cell) and showing a larger amplitude of Ca^2+^-signals during immobility than during locomotion (*p* < 0.01, one-way ANOVA/Tukey’s test)

To understand the functional role of VIP-LRP cells, we performed in vivo two-photon calcium (Ca^2+^) imaging of VIP+ interneuron activity in head-restrained awake mice running on a treadmill (Fig. 5a–d)^39^. The *Cre*-dependent viral vector adeno-associated virus (AAV)1.Syn.Flex.GCaMP6f.WPRE.SV40 was delivered to the CA1 hippocampus of *VIP-Cre* mice to express Ca^2+^-sensitive protein GCaMP6f selectively in VIP+ neurons (Fig. 5b). The immunohistochemical analysis of VIP+ O/A neurons in VIP-*Cre* mice (Supplementary Fig. 3a–d) confirmed that VIP/M2R-co-expressing cells were present in the CA1 O/A (albeit at a significantly lower fraction when compared with VIP-eGFP mice: 7% in *VIP-Cre* vs 50% in VIP-GFP out of total VIP+ O/A cells; Supplementary Figs. 2i and 3e–h), likely due to the mouse strain differences (CD1 for VIP-eGFP vs C57BL/6J for *VIP-Cre* mice)^40–42^. The M2R+ VIP O/A cells in *VIP-Cre* mice exhibited virus-driven GCaMP6f expression, showed a normal morphological appearance (Fig. 5e) and were examined for the activity-dependent recruitment during different behavioural states.

**Fig. 6:**
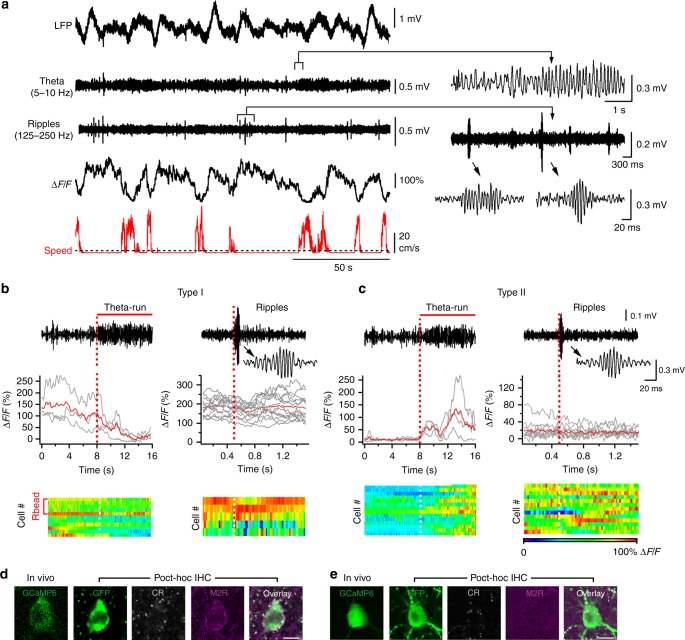
Network state-dependent recruitment of VIP-OA interneurons in awake mice. **a** Representative traces of simultaneous LFP (raw trace and filtered for theta and ripples) and Ca^2+^-transient (Δ*F*/*F*) recordings from a putative VIP-LRP cell identified post hoc as M2R-positive (**d**). Red trace illustrates the animal locomotion speed (dotted line indicates the threshold for the locomotion state at 2 cm/s). **b** Individual traces from the event-triggered Ca^2+^-trace segmentation and corresponding average (red trace) generated by the theta-run epochs (left) and ripple episodes (right; with inset showing an expanded view of the ripple event) from the cell illustrated in **a** with heat-maps showing the group data for all VIP-LRPs (*n* = 33 events/11 cells for theta-run; *n* = 56 events/5 cells for ripples). The decrease in somatic Ca^2+^-signals was significant during theta-run epochs for a group of cells (*n* = 6) at *p* < 0.001; Mann–Whitney test. **c** Representative traces from the event-triggered Ca^2+^-trace segmentation with corresponding average (red trace) generated by the theta-run epochs (left) and ripples (right) with heat-maps showing the group data for type II M2R-/CR- VIP-expressing cells (*n* = 14 cells for theta-run; *n* = 13 cells for ripples). Increase in somatic Ca^2+^-signals during theta-run epochs was significant for a group of cells (*n* = 14) at *p* < 0.001; Mann–Whitney test. **d** Post hoc immunohistochemical analysis of the recorded VIP-LRP showing that cells of this sub-type (type I) express M2R. GFP was revealed with Alexa-488, CR with Cy3 and M2R with CF-633 secondary antibodies. Scale bar: 10 µm. **e** Post hoc immunohistochemical analysis of the recorded type II VIP-expressing cells showing that cells of this type do not express M2R or CR. Scale bar: 10 µm

During the experiment, habituated mice showed spontaneous alternations in their behaviour between locomotion (running speed median and interquartile range: 8.8 and 5–30 cm/s; Fig. 5c), immobility and flickering^39^, a transitional state associated with brief random movements (Fig. 5a, *n* = 6 mice). As flickering periods were very short (<1 s) and variable in occurrence, they were excluded from further analysis. Among 54 CA1 VIP+ interneurons imaged, 28 VIP+ cells were located within O/A and analyzed in detail during 409 locomotion and 356 immobility periods (10–15 periods/cell pooled from two independent imaging sessions of 5 min each/3 mice; Table S4). Consistent with previous observations in neocortical circuits^13^, as a population, the majority of VIP+ O/A cells were more active during animal locomotion (Supplementary Table 4). By applying Otsu’s method^43^ to somatic Ca^2+^-activity, we could segregate these cells into two distinct sub-types (Fig. 5d): type I VIP cells (*n* = 8), on average, exhibited higher somatic Ca^2+^-signals during immobility than during locomotion (locomotion: 30.0 ± 13.2% Δ*F*/*F* vs immobility: 100.4 ± 28.0% Δ*F*/*F*; *p* < 0.05; Mann–Whitney test; Fig. 5d), while type II cells (*n* = 20) were more active during locomotion than during quiet states (locomotion: 94.9 ± 10.1% Δ*F*/*F* vs immobility: 31.7 ± 4.0% Δ*F*/*F*; *p* < 0.001; Mann–Whitney test; Fig. 5d). Post hoc immunohistochemical analysis of recorded neurons (6 out of 28 VIP+ O/A interneurons recorded in vivo were found after brain re-sectioning and processed for markers; Fig. 5e) revealed that type I cells which were more active during quiet states express M2R but not CR (2 cells out of 2 tested) and, therefore, correspond to VIP-LRP neurons (Figs. 5e and 6d). The type II VIP cells tested were negative for both M2R and CR (4 cells out of 4 tested; Fig. 6e) and, therefore, could correspond to VIP-BCs or other VIP+ interneurons.

To further validate these data, we performed Ca^2+^ imaging of CA1 O/A VIP+ interneurons that were retrogradely labelled with red RetroBeads injected in SUB in addition to *Cre*-driven GCaMP6f expression (Fig. 5f; *n* = 5 cells/2 mice). Local delivery of a small volume of RetroBeads in SUB (25 nL; Fig. 5f, left top) resulted in labelling of CA1 PCs as well as of a few O/A interneurons, which is consistent with previous observations of the several distinct types of SUB-projecting GABAergic cells in the CA1 O/A^27,29,33^ (Fig. 5f, left bottom). Out of total 11 interneurons labelled with RetroBeads, 5 were VIP+ cells, which thus corresponded to VIP-LRPs (Fig. 5f, right). We confirmed that bead-labelled interneurons exhibit normal physiological properties using patch-clamp current clamp recordings in vitro (Supplementary Fig. 4a, 4b). In vivo, Ca^2+^ transients detected in retrogradely labelled VIP-LRPs had larger peak amplitude during immobility than during locomotion periods (immobility: 55.5 ± 6.7% Δ*F*/*F*, *n* = 81 periods/5 cells; locomotion: 21.0 ± 3.2% Δ*F*/*F*, *n* = 63 periods/5 cells; *p* < 0.01, one-way ANOVA followed by Tukey’s test; Fig. 5g; Supplementary Fig. 4c, d). Taken together, these data indicate that VIP-LRPs are more active at rest than during locomotion.

As VIP-LRP cells showed different levels of somatic activity during behavioural states, we next examined their recruitment during network oscillations through parallel recordings of the local field potential (LFP) from the contralateral CA1 hippocampus^39,44^. The locomotion periods were associated with prominent theta oscillations (7.1 ± 0.3 Hz; *n* = 6 mice; Fig. 6a–c), while high-frequency ripples were observed during the animal quiet state (144.5 ± 2.6 Hz; *n* = 6 mice; Fig. 6a–c). For VIP-LRP population (type I VIP+ cells), we combined the cells segregated based on their behaviour activity pattern (*n* = 6 cells with LFP recorded; Fig. 5d) with those that were labelled retrogradely (*n* = 5; Fig. 5f, g). In these cells, the peak somatic Ca^2+^ signal decreased significantly, from 80.80 ± 9.03% Δ*F*/*F* during quiet states to 38.61 ± 5.15% Δ*F*/*F* during theta-run epochs (*p* < 0.001; Mann–Whitney test; 154 stationary and 116 theta-run periods, *n* = 11 cells; Fig. 6b), indicating that VIP-LRPs decrease their activity during theta oscillations. In contrast, the M2R-/CR- type II VIP+ cells increased their activity from 31.8 ± 3.3% Δ*F*/*F* during quiet state to 96.8 ± 6.1% Δ*F*/*F* during theta-run episodes (*p* < 0.001; Mann–Whitney test; 162 stationary and 190 theta-run periods, *n* = 14 cells; Fig. 6c), pointing to the on-going recruitment of these cells during theta. As the quiet state in awake rodents is associated with recurrent ripple oscillations^2,45,46^ and these events may co-occur in the two hippocampi^44,47–49^ (but see recent findings for rats during sleep^50^), we investigated the potential recruitment of VIP+ neurons during ripple episodes. The VIP-LRPs showed no change in somatic activity in relation to ripple episodes (57.2 ± 34.1% Δ*F*/*F* before vs 52.7 ± 29.3% Δ*F*/*F* after ripple episode, *n* = 56 episodes/5 cells; *p* > 0.05; Mann–Whitney test; Fig. 6b). Similar data was obtained for the type II M2R-/CR- VIP+ cells (22.2 ± 4.5% Δ*F*/*F* before vs 23.5 ± 6.2% Δ*F*/*F* after ripple episode, *n* = 13; *p* > 0.05; Mann–Whitney test; Fig. 6c), although strong ripple coupling was observed in some M2R-/CR- VIP-expressing cells located within PYR (Supplementary Fig. 5). Collectively, these data reveal the preferential recruitment of VIP-LRPs during quiet wakefulness and the suppression in their activity during theta-run epochs, pointing also to functional segregation of VIP interneuron sub-types during different behavioural and network states.

### Diversity of subiculum-projecting VIP-LRPs

**Fig. 7:**
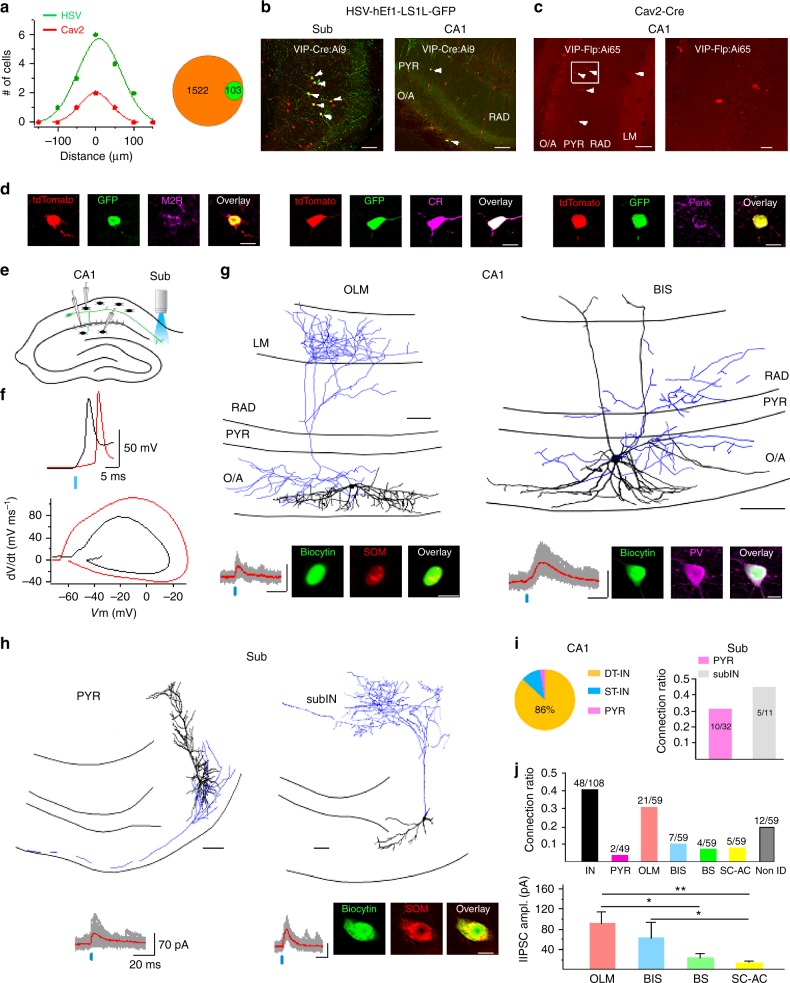
Cellular diversity and connectivity of subiculum-projecting VIP-LRPs. **a** Summary distributions (left) of infected neurons within SUB for HSV-hEf1-LS1L-GFP and Cav2-*Cre* retrograde viruses following injection in SUB (the ‘zero’ distance indicates the virus injection focus; data points indicate the number of cells targeted in the adjacent slices), and a pie-chart (right) illustrating the VIP-LRP fraction (green) out of the total VIP+ population (orange) in the CA1 area of VIP-*Cre*;Ai9 mice using a Cre-inducible herpes simplex virus (HSV-hEf1-LS1L-GFP). **b** Representative images showing retrograde labelling of VIP-LRPs using HSV-hEf1-LS1L-GFP in *VIP-Cre;Ai9* mice (left). Retrogradely labelled GFP/tdTomato-coexpressing VIP-LRPs (indicated with white arrowheads) were found within subiculum (left) and CA1 (right). Scale bar: 100 µm. **c** Combinatorial genetic labelling of VIP-LRPs using Cav2-*Cre* injections in the subiculum of *VIP-Flp;Ai65* mice. White arrowheads point to VIP-LRPs expressing tdTomato under *cre* and *flp* control. The area squared on the left is expanded on the right. Scale bar: 20 µm. **d** Representative confocal images illustrating the VIP-LRP markers in *VIP-Cre;Ai9* mice: M2R (left), CR (center) and Penk (right). Scale bars: 10 µm. **e** Schematic illustration of the optogenetic VIP-LRP activation through light stimulation in the subiculum. **f** Example traces and summary phase plots for antidromic spike (red) evoked in VIP-LRP by light stimulation vs somatically evoked spike (top, black). **g**, **h** Light-evoked IPSCs in response to antidromic VIP-LRP activation in different CA1 (**g**) and subicular (**h**) targets, including an O-LM (**g**, left) and a BIS (**g**, right) cells in the CA1 area, as well as pyramidal cells (**h**, left) and a SOM-positive interneuron (**h**, right) in subiculum (scale bars: 100 µm for reconstructions, 20 µm (**g** bottom), 10 µm (**h**, bottom)). **i** Pie-chart (left) illustrating the distribution of VIP-LRP CA1 targets (DT-IN dendrite-targeting interneuron, ST-IN soma-targeting interneuron, PYR pyramidal cell) and summary bar graphs (right) showing the connection ratio for subicular targets (PYR pyramidal cell, subIN subicular interneuron). **j** Summary bar graphs illustrating the connection ratio and the lIPSC amplitude (mean ± SEM) for CA1 targets (O-LM *n* = 18, BIS *n* = 5, BC *n* = 4, SC-AC *n* = 4; O-LM vs Bis, *p* > 0.05, O-LM vs BC, *p < 0.05, O-LM vs SC-AC, **p < 0.01, Mann–Whitney test)

How diverse is the population of SUB-projecting VIP-LRPs? To address this question, we conducted retrograde tracing by (1) injecting a small volume (20 nL) of *Cre*-dependent hEf1-LS1L-GFP herpes simplex virus (HSV) into the SUB of *VIP-Cre;Ai9* mice (Fig. 7a, b) or (2) using a combinatorial VIP-LRP targeting via injection of retrograde Cav2-*Cre* into SUB of *VIP-flp;Ai65* mice (Fig. 7a, c). The reporter Ai65D (B6;129S-Gt(ROSA)26Sortm65.1(CAG-tdTomato)Hze/J) mouse line expresses tdTomato under the control of *cre* and *flp*. Crossing this reporter line with *VIP-flp* mice and injecting the Cav2-*Cre* in the SUB allows selective targeting of VIP+ SUB-projecting neurons. With both strategies, prior calibration experiments were performed to control for the virus spread from SUB to CA1 (Fig. 7a; see Methods for details). In addition to a small population of local SUB VIP+ cells (Fig. 7b, left), CA1 VIP+ interneurons with somata located within O/A, PYR, RAD or LM were sparsely labelled (*VIP-Cre;Ai9* mice + HSV-GFP: 6.7 ± 0.4% of total CA1 VIP+ population, 103/1522 cells from 3 animals, Fig. 7a, b; *VIP-flp;Ai65* + Cav2-*Cre*: 7.3 ± 0.6% of total CA1 VIP+ population, 100/1364 cells from 3 animals, Fig. 7a, c). O/A VIP-LRPs made ~20% of the total VIP-LRP population (22 out of 103 cells in *VIP-Cre;Ai9* mice). Consistent with our findings of a low fraction of M2R+ VIP O/A cells in *VIP-Cre* mice (Supplementary Fig. 3e), some O/A VIP-LRPs labelled with an HSV-GFP in *VIP-Cre;Ai9* mice co-expressed M2R (Fig. 7d, left; 2 out of 13 O/A VIP-LRPs tested). In addition, those with soma located within PYR, RAD or LM co-expressed CR (10 out of 31 cells tested; Fig. 7d, middle) or proenkephalin (Penk, 2 out of 28 cells tested; Fig. 7d, right), revealing further molecular diversity within the SUB-projecting VIP-LRP population.

To examine the local connectivity of the entire SUB-projecting VIP+ population in the CA1 area, we next employed a ChR2-assisted circuit mapping approach based on the antidromic activation of VIP-LRP cells through wide-field stimulation of their axons in the SUB of *VIP-Cre;Ai32* mice (Fig. 7e, f). Importantly, we found no evidence for the existence of subiculo-hippocampal VIP+ projecting neurons that could be activated by light-stimulation in SUB and contact CA1 interneurons (Supplementary Fig. 6). In addition, no antidromic spikes were evoked in VIP-BCs (*n* = 3) or IS3 cells (*n* = 3) by light stimulation in SUB (data not shown), thus validating our photostimulation approach for antidromic activation of hippocampo-subicular VIP-LRP neurons.

In total, 59 CA1 interneurons and 49 CA1 PCs were examined as potential VIP-LRP targets. In CA1 O/A, 33 out of 36 interneurons tested were connected and 26 were visualized with biocytin (Fig. 7g), including O-LM (*n* = 21) and BIS (*n* = 7) cells (Fig. 7g). Moreover, a putative LRP cell, that was negative for SOM and M2R, with a partially myelinated axon travelling outside the hippocampus, received inhibitory input from VIP-LRP neurons (Supplementary Fig. 7a). In CA1 RAD, 12 out of 23 interneurons tested received input from VIP-LRPs, including CCK-expressing Schaffer-collateral-associated cells (*n* = 5) and BCs (*n* = 4; Supplementary Fig. 7b, c). The amplitude of light-evoked IPSCs (lIPSCs) was substantially higher in O/A (88. 6 ± 18.3 pA, *n* = 26) than in RAD interneurons (30.5 ± 7.6 pA, *n* = 8; *p* < 0.01, Mann–Whitney test), with the SOM+ O-LM and BIS cells demonstrating the largest amplitude of lIPSCs. In contrast, out of 49 PCs tested, only 2 cells with soma within O/A received input from VIP-LRPs (Supplementary Fig. 7d, e). These data further support the preferential interneuron innervation by VIP-LRP population, revealing the CA1 circuit disinhibition as a local function of SUB-projecting VIP-LRPs.

Subicular targets were also examined using a ChR2-assisted mapping strategy but through wide-field photostimulation in CA1 and patch-clamp recordings in subiculum. Out of 43 attempts, 15 subicular neurons were connected to VIP-LRPs (Fig. 7h, i). Morphological analysis of cells filled with biocytin showed that SUB targets of VIP-LRPs included both PCs (*n* = 10 out of 32 attempts; Fig. 7h, left; i) and interneurons (*n* = 5 out of 11 attempts; one was identified as SOM+, Fig. 7h, right; i), thus pointing to a shared VIP-LRP input by PCs and interneurons in the distant projection area (Fig. 7j). Taken together, these data highlight the region-specific target preference of VIP-LRP population and suggest their potential functional role in setting up CA1 disinhibition concurrently with an inhibitory reset in the subiculum.

## Discussion

We discovered a novel population of hippocampal VIP-expressing GABAergic neurons that exhibit specific molecular properties and, in addition to local innervation of CA1, also make long-range projections to the subiculum with region-specific connectivity patterns. These cells are only weakly active during theta oscillations associated with locomotion but maintain high activity level during quiet state. The latter may promote disinhibition of CA1 PCs in parallel with inhibition–disinhibition periods in the subiculum due to a concomitant innervation of both subicular PCs and interneurons. The likely role of VIP-LRP neurons is therefore to synchronize PC ensembles along the hippocampo-subicular axis that may be necessary for memory consolidation during animal quiet state.

We show that VIP-expressing neurons in the mouse CA1 hippocampus form several functionally and molecularly distinct populations, including BCs, local circuit IS cells and LRP neurons. The VIP-LRP cell is a novel circuit element to be included in the CA1 connectome. Here, we focused on O/A VIP-LRPs, which correspond to ~20% of the total VIP-LRP population. This cell type is different from IS3 interneurons, which express CR, exhibit distinct electrophysiological parameters and have a similar axon distribution within the CA1 but target local inhibitory neurons^24,38^. Indeed, the major and, perhaps, the most striking feature of VIP-LRPs is their distant projection and innervation of both interneurons and PCs in the distal projection area.

We demonstrate that, locally, VIP-LRP neurons prefer to make synapses with different classes of inhibitory interneurons, either in the O/A or in the RAD. Interneurons that are known to innervate the PC dendrites, including the O-LM, the BIS and the SC-AC cells, were among the targets of VIP-LRP axons. In addition, perisomatic terminating BCs were also innervated. As the activity of VIP-LRPs was strongly decreased during theta-run epochs, these cells are unlikely to modulate CA1 interneuron firing during theta oscillations associated with locomotion. Indeed, in agreement with our observations, most interneurons exhibit a time-locked maximal activity during theta oscillations necessary for the temporal sequence representation in PC firing^2,45,51,52^. Interestingly, Buzsáki et al.^31^ identified some rare cells in the hilus as ‘anti-theta’ cells, which were later found in the CA1, subiculum and entorhinal cortex, and classified as theta-off cells^32,53,54^. Despite the remarkable network behaviour of theta-off cells, their cellular identity and connectivity patterns have remained unknown. We provide evidence that, at least in the CA1 hippocampus, theta-off cells include a population of VIP-LRP GABAergic neurons that mediate local disinhibition. Importantly, the theta-off cells can display tonic firing by inactivation of the MS^54^, pointing to critical MS suppressive influences in their network motif. In particular, the activation of the M2Rs expressed in the somato-dendritic membrane of these cells or at presynaptic excitatory terminals may be responsible for suppression in VIP-LRP activity during theta oscillations^55–57^. Furthermore, the activation of local or long-range GABAergic projections^7,58,59^ that likely converge onto VIP-LRP neurons may prevent these cells from firing during theta-run epochs. Our data also indicate that, in response to the theta-modulated input, VIP-LRPs show weak synchrony due to surround inhibition resulting from the preferential propagation of the spike AHP through gap junctions^60^. Therefore, on the contrary to other cell types^61,62^, electrotonic coupling between VIP-LRPs does not promote their synchrony; at least this is not the case in response to the theta-like input. How the different firing frequency, the state of gap junctions or the number of coupled neurons participating in the network activity^35–37^ may shape the cell recruitment remains to be determined.

Are VIP-LRPs discovered here similar to other subiculum-projecting hippocampal GABAergic neurons? One candidate is the trilaminar cell identified previously in the rat hippocampus^63^, which was shown to express M2R in the somato-dendritic membrane, was decorated with mGluR8a-containing terminals and projected to the subiculum^33^. This cell had a large soma with horizontally running dendrites at the O/A border and an axon innervating the CA1 from O/A through PYR to RAD. However, the distribution of the trilaminar cell axon in the rat was biased toward proximal RAD (~70%^63^), which is not the case for VIP-LRP cells in the mouse. The presence of VIP has not been reported in the trilaminar cell, and, in contrast to VIP-LRPs, this cell shows complex spike bursts during theta oscillations and strong discharges during ripples^33^. The other populations of subiculum-projecting GABAergic neurons were described in the outer molecular layer of the dentate gyrus^64^ and in the CA1^27,29^. The latter express COUP-TFII alone or in combination with enkephalin or calretinin, but do not express VIP. Moreover, in contrast to VIP-LRPs, these subiculum-projecting GABAergic neurons are strongly modulated during theta oscillations.

In conclusion, our data identify the VIP-LRP neuron as a novel circuit element, which, through its region- and target-specific GABAergic interactions, controls the information flow along the hippocampo-subicular axis. As activation of VIP-LRPs occurred preferentially during animal quiet state, this cell type constitutes a good candidate for hippocampo-subicular mnemonic processing associated with episode recollection and comparison^65,66^. Indeed, the coherency between the two regions increases during quiet network states^67,68^ and, in addition to other mechanisms, may require the involvement of hippocampo-subicular VIP-LRP GABAergic neurons.

## Methods

### Mouse lines

Nine mouse lines were used in this study: the previously characterized VIP/enhanced green fluorescent protein (VIP-eGFP^24^); mice [BAC line with multiple gene copies; MMRRC strain #31009, STOCK Tg(Vip-EGFP) 37Gsat, University of California, Davis, CA]; the previously described *VIP*-*Cre* mice (stock #010908, The Jackson Laboratory^69^); the previously characterized *VIP-Cre;Ai32* mice^70^, which were obtained by breeding the *VIP-Cre* with the Ai32 line (B6;129S-Gt(ROSA)26Sortm32(CAG-COP4*H134R/EYFP)Hze/J; stock #012569, The Jackson Lab); *Vip-Cre;Ai9* mice obtained by breeding the *VIP-Cre* mice with the reporter line Ai9-(RCL-tdTomato)line(B6.Cg-Gt(ROSA)26Sor^tm9(CAG-tdTomato)Hze^/J, stock #007909, The Jackson Laboratory) and *Vip-Flp;Ai65* mice obtained by breeding the *VIP-FlpO* mice (kindly provided by Dr. Ed Callaway under agreement with Dr. Josh Huang, CSHL) with a combinatorial reporter Ai65D line (B6;129S-Gt(ROSA)26Sortm65.1(CAG-tdTomato)Hze/J, stock #021875, The Jackson Laboratory). In VIP-eGFP mice, virtually all interneurons that were immunoreactive for VIP endogenously were confirmed to express eGFP (Supplementary Fig. 2g, i; see also ref. ^24^). In *VIP-Cre;Ai9* mice hippocampus (Supplementary Fig. 3), we also confirmed the presence of molecular cell type markers found in VIP-eGFP mouse interneurons, albeit at a different proportion. Mice had access to food and water ad libitum and were housed in groups of two to four. Mice of either sex, of CD1 and C57BL/6J genetic backgrounds, at postnatal days 19–50 were used for all experiments. Mice were randomly assigned to experimental groups, which were matched in terms of numbers of males and females in each group. Mice undergoing surgery were housed separately (1/cage). All experiments were approved by the Animal Protection Committee of Université Laval and the Canadian Council on Animal Care. The minimal number of animals necessary for the appropriate sample size was used.

### Viral constructs

The pAAV-Ef1a-DIO-hChR2(H134R)-EYFP-WPRE-pA virus was acquired from the University of North Carolina (UNC) at Chapel Hill Vector Core. The AAV1.Syn.Flex.GCaMP6f.WPRE.SV40 was acquired from the University of Pennsylvania Vector Core. The hEf1-LS1L-GFP HSV vector was provided by Dr. Rachael Neve at the MIT Viral Gene Transfer Core and packaged at the University of Massachusetts Medical School Gene Therapy Center and Vector Core. The Cav2-*Cre* virus was acquired from the Plateforme de Vectorologie de Montpelier (PVM) at Bio-Campus Montpelier.

### Slice preparation and patch-clamp recordings

Transverse hippocampal slices (thickness, 300 µm) were prepared from VIP-eGFP or *VIP-Cre;Ai32* mice of either sex as described previously^24,38^. Briefly, animals (P15-30) were anaesthetized deeply with isoflurane or ketamine–xylazine (ketamine: 100 mg/kg, xylazine: 10  mg/kg) and decapitated. The brain was dissected carefully and transferred rapidly into an ice-cold (0–4 °C) solution containing the following (in mM): 250 sucrose, 2 KCl, 1.25 NaH_2_PO_4_, 26 NaHCO_3_, 7 MgSO_4_, 0.5 CaCl_2_ and 10 glucose oxygenated continuously with 95% O_2_ and 5% CO_2_, pH 7.4, 330–340 mOsm/L. Transverse hippocampal slices (thickness, 300 µm) were cut using a vibratome (VT1000S; Leica Microsystems or Microm; Fisher Scientific), transferred to a heated (37.5 °C) oxygenated recovery solution containing the following (in mM): 124 NaCl, 2.5 KCl, 1.25 NaH_2_PO_4_, 26 NaHCO_3_, 3 MgSO_4_, 1 CaCl_2_ and 10 glucose; pH 7.4; 300 mOsm/L and allowed to recover for 1 h. Subsequently, they were kept at room temperature until use. During experiments, slices were continuously perfused (2 mL/min) with standard artificial cerebrospinal fluid (ACSF) at physiological temperature (30–33 °C) containing the following (in mM): 124 NaCl, 2.5 KCl, 1.25 NaH_2_PO_4_, 26 NaHCO_3_, 2 MgSO_4_, 2CaCl_2_ and 10 glucose, pH 7.4 saturated with 95% O_2_ and 5% CO_2_. VIP-positive O/A interneurons were visually identified as GFP-expressing cells upon illumination with blue light (filter set: 450–490 nm). Two-photon images of GFP-expressing interneurons in acute slices were obtained using a two-photon microscope (TCS SP5; Leica Microsystems) based on a Ti-Sapphire laser tuned to 900 nm. Images were acquired with a 25× water-immersion objective (NA 0.95). Whole-cell patch-clamp recordings were obtained from single cells or pairs of neurons in voltage- or current-clamp mode. Recording pipettes (3.5–6 MΩ) were filled with a Cs-based solution for voltage-clamp recordings (in mM): 130 CsMeSO_4_, 2CsCl, 10 diNa-phosphocreatine, 10 HEPES, 4 ATP-Tris, 0.4 GTP-Tris, 0.3% biocytin, 2 QX-314, pH 7.2–7.3, 280–290 mOsm/L; or a K^+^-based intracellular solution for current-clamp recordings (in mM): 130 KMeSO_4_, 2 MgCl_2_, 10 diNa-phosphocreatine, 10 HEPES, 4 ATP-Tris, 0.4 GTP-Tris and 0.3% biocytin (Sigma), pH 7.2–7.3, 280–290 mOsm/L. Data acquisition (filtered at 2–3 kHz and digitized at 10 kHz; Digidata 1440, Molecular Devices, CA, USA) was performed using the Multiclamp 700B amplifier and the Clampex 10.5 software (Molecular Devices). Active membrane properties were recorded in current-clamp mode by subjecting cells to multiple current step injections of varying amplitudes (−240 to +280 pA).

To assess synaptic connectivity between VIP-LRPs and O/A interneurons, two neurons were recorded simultaneously, with the presynaptic interneuron (VIP-LRP) kept in current-clamp mode at −60 mV and the postsynaptic cell (O/A interneuron) held in voltage-clamp mode at 0 mV. The junction potential was not corrected. APs were evoked in the presynaptic interneuron via two brief somatic current injections (2 ms, 1–1.5 nA) at 20 Hz. In case of synaptic connection, this protocol evoked short-latency (<5 ms) unitary IPSCs (uIPSCs) in the postsynaptic cell. Although individual uIPSCs were small (~10 pA) and close to the noise level in our experiments (5–9 pA), we could detect them based on a constant latency (Fig. 2c, f, g). The pipette capacitance and series resistance (in voltage-clamp configuration) were compensated and bridge balance (in current-clamp configuration) was adjusted. The series resistance (*R*ser) before compensation was 15–20 MΩ and was monitored continuously by applying a −5 mV step at the end of every sweep. Recordings with changes in *R*ser > 15% were removed from the analysis. To detect changes in uIPSCs amplitude during different frequencies of firing of VIP-LRPs, APs were generated in VIP-LRPs at 10, 50 and 100 Hz. To examine electrical coupling between VIP-LRPs, two neurons were recorded simultaneously as mentioned above in the presence of synaptic blockers: gabazine (1 µM), NBQX (10 µM) and AP5 (100 µM). The coupling coefficient (CC_12_) was calculated as the ratio of voltage responses of the receiving cell (here, cell 2) to the stimulated cell (here, cell 1) with a hyperpolarizing current step (−140 pA, 1000 ms) applied to the cell 1. Pairs were considered to be electrically coupled if their coupling coefficient was ≥0.01. Gap junctions were tested with a connexin-36, connexin-50 and connexin-43 gap-junction blocker mefloquine (100 µM, #M2319, Sigma) or a broad-spectrum gap-junction blocker carbenoxolone (100 µM, #C4790, Sigma). A sinusoidal excitatory input modulated at theta-frequency (5-Hz) was applied to the electrically coupled pair in three different conditions: to the cell 1 only with both cells held at resting membrane potential (Fig. 3f), to the cell 1 only when cell 2 was depolarized to allow for spontaneous firing (Fig. 3g), and to both cells at more depolarized membrane potential (Fig. 3h).

### Two-photon laser scanning photostimulation by glutamate uncaging

Two-photon glutamate uncaging experiments were performed as described previously^38^. Briefly, acute hippocampal slices (300 µm) were obtained from VIP-eGFP mice (P15–25) and perfused during experiment with ACSF containing high Ca^2+^ (4 mM), high Mg^2+^ (4 mM) and DL-AP5 (50 μM) to reduce the spontaneous synaptic activity and the confounding polysynaptic effects. To avoid non-specific effects of 4-methoxy-7-nitroindolinyl (MNI)-caged glutamate (Glu) on inhibitory synaptic transmission, the MNI-Glu (5 mM; Tocris) was applied locally by fast micropressure pulses (5 psi, 5 ms) via a glass pipette (tip diameter of 2–3 μm) connected to a pressure application system (PicoSpritzer II; Parker Instrumentation, Fairfield, NJ, USA) and positioned ∼10 μm above the putative VIP-LRP. The putative VIP-LRPs were selected for photoactivation based on their soma location in the CA1 O/A and expression of eGFP. They were visualized for puff-pipette positioning and two-photon somatic glutamate uncaging with a two-photon Dodt infrared scanning gradient contrast technique (Dodt-IRSGC^38^) using a two-photon laser scanning system (Leica TCS SP5 microscope with a 40×, 0.8 NA water-immersion objective; Leica Microsystems) based on a Ti-Sapphire laser tuned to 730 nm (laser power measured under the objective, 5–10 mW). Focal release of glutamate was accomplished by illuminating the somatic region for ~180 ms (laser power, 25–30 mW) immediately after puff application of the caged compound. These settings were reliable in evoking a single spike in VIP+ O/A interneurons. To prevent photodamage, the stimulations were repeated once every 30 s and the laser power did not exceed 40 mW (measured under the objective). Control experiments included the application of MNI-Glu without subsequent uncaging, and uncaging without prior application of MNI-Glu^38^.

### ChR2-based mapping of VIP-LRP targets

Optogenetic activation of VIP-LRPs was achieved through wide-field low-intensity stimulation with blue light (filter set: 450–590 nm; average power at the sample, 1.30 mW; pulse duration 2.5 or 5 ms, which was corresponded to the minimal duration able to evoke the response) using a 40× water-immersion objective (NA 0.8), which was applied to an area of ~0.2 mm^2^ within the SUB 1.0–1.2 mm away from the CA1 border to generate antidromic spikes in VIP-LRPs while avoiding the activation of VIP-BCs and IS3 cells in CA1. The generation of antidromic spikes was confirmed using current-clamp recordings from VIP-positive O/A neurons in *VIP-Cre;Ai32* mice and photostimulation in SUB. The antidromic spike generated in this case differed from the somatic one evoked by light illumination in the CA1 area (Fig. 7f). The opposite experimental paradigm, with patch-clamp recordings in the SUB and photostimulation in the CA1, was applied to investigate the targets of VIP-LRPs in subiculum. The light-evoked IPSCs (IPSCLs) were recorded at 0 mV. Using a low-light stimulation paradigm allowed for spatially localized excitation, which generated both successful lIPSCs and failures (Fig. 7g, h; Supplementary Fig. 7a, c, d).

### In vitro patch-clamp data analysis

Analysis of electrophysiological recordings was performed using Clampfit 10.6 (Molecular Devices) and Igor Pro 6.2 (WaveMetrics). For the analysis of the AP properties, the first AP appearing at current pulse of +40 to 60 pA within a 50-ms time window was analyzed. The AP amplitude was measured from the threshold to the peak. The AP latency was measured from the beginning of the current pulse to the AP threshold level. The AP half-width was measured at the voltage level of the half of AP amplitude. The fast AHP amplitude was measured from the AP threshold. *I*_h-_associated voltage rectification was determined as the amplitude of the membrane potential sag from the peak hyperpolarized level to the stable level when hyperpolarized to −100 mV.

To analyze the properties of uIPSCs, 100 sweeps were acquired. Sweeps with spontaneous activity occurring right before or during uIPSCs were removed. The failures were identified from individual sweeps as traces that did not contain any time-dependent signal after the end of the presynaptic AP. The failure rate was calculated as the number of failures divided by the total number of traces. After this step, all sweeps containing failures were removed and successful uIPSCs were averaged to obtain uIPSC potency for further analysis. The uIPSC latency was determined as the time interval between the peak of a presynaptic AP and the onset of the uIPSC in the postsynaptic cell. The rise time of uIPSC was taken at 20–80% and a monoexponential decay time constant was determined. We did not attempt to calculate the uIPSC synaptic conductance since the GABA reversal potential can be different at different targets and was not examined in this study. The paired-pulse ratio was determined as the ratio between the mean peak amplitude of the second response and the mean peak amplitude of the first response, which were obtained 50 ms apart, including failures. During repetitive stimulation (10–100 Hz; Fig. 2c), the peak amplitudes of individual uIPSCs were extrapolated from the baseline by fitting the decay of the preceding uIPSC at the average trace. The connection ratio for specific postsynaptic targets (OLM, BIS and BC; Fig. 2i) was determined as a ratio between the number of connected cells of a specific type to the total number of recording attempts (*n* = 118).

For ChR2-based mapping analysis, the potency of the light-evoked IPSCs (lIPSCs) was determined as the average lIPSC obtained after removal of all sweeps containing failures. The connection ratio for each specific target was determined as described above for paired recordings.

For electrical coupling analysis, cross-correlation functions in Clampfit were used to explore synchrony in voltage fluctuations (Fig. 3f) or firing (Fig. 3g, h) between electrically coupled VIP-LRPs. For spike synchrony, cross-correlation analysis was performed on high-pass (at 125 Hz) filtered voltage traces following the spike detection algorithm to correlate spike start times between the two connected cells.

### Cell reconstruction and immunohistochemistry

For post hoc reconstruction, neurons were filled with biocytin (Sigma) during whole-cell recordings. Slices with recorded cells were fixed overnight with 4% paraformaldehyde (PFA) at 4 °C. To reveal biocytin, the slices were permeabilized with 0.3% Triton X-100 and incubated at 4 °C with streptavidin-conjugated Alexa-488 or Alexa-546 (1:1000) in TBS. For combined morphological and immunohistochemical analysis of recorded cells, the duration of whole-cell recordings was reduced to 10 min, and the concentration of biocytin was increased to 0.5% for reliable axonal labelling. This procedure was not required for the analysis of the expression of the membrane-bound proteins (e.g. M2R, mGluR1).

All immunohistochemical tests were performed on free-floating sections (40 or 70 µm thick) obtained with Leica VT1000S or PELCO EasySlicer vibratomes from 3–4 mice (20 sections/animal) per condition. VIP-eGFP, VIP-*Cre* (injected with GCaMP6f) or VIP-*Cre*;Ai9 mice were perfused with 4% PFA and the brains were sectioned. Sections were permeabilized with 0.25% Triton X-100 in PBS and incubated overnight at 4 °C with primary antibodies followed by the secondary antibodies. The list of primary and secondary antibodies used is provided in Supplementary Table 2. For proenkephalin immunoreaction, biotinylation was performed to enhance the labelling specificity. Briefly, following overnight incubation of sections with rabbit proenkephalin primary antibody, biotinylated anti-rabbit antibody was applied for 24 h followed by streptavidin-conjugated AlexaFluor (1:1000; Supplementary Table 2). For controlling method specificity, the primary antibodies were omitted and sections incubated in the full mixture of secondary antibodies. Under such conditions no selective cell labelling was detected. Confocal images were acquired sequentially using a Leica TCS SP5 imaging system coupled with a 488-nm argon, a 543-nm HeNe and a 633-nm HeNe lasers. Z-stacks of biocytin-filled cells were acquired with a 1-μm step and merged for detailed reconstruction in Neurolucida 8.26.2. The axon length was measured without shrinkage correction. For Fig. 1f, an LSM710 confocal microscope (Axio Imager.Z1, Carl Zeiss) with ZEN 2008 software v5.0 (Zeiss) was used to acquire multi-channel fluorescence images sequentially with a DIC M27 Plan-Apochromat 63× (NA 1.4) objective. The cells were considered immunopositive when the corresponding fluorescence intensity was at least twice of that of the background. For representation only, the overall brightness and contrast of images were adjusted manually. Portions of images were not modified separately in any way. As the antibody to detect immunoreactivity for mGluR8 is sensitive to fixation conditions, we used sections from one well-reacting mouse.

### Retrograde labelling

VIP-eGFP, *VIP-Cre;Ai9* or *VIP-flp;Ai65* mice (P30–100) were anesthetised deeply via the intraperitoneal injection of ketamine/xylazine (ketamine: 100 mg/kg, xylazine: 10 mg/kg). After receiving a subcutaneous injection of Buprenorphine SR (0.6 mg/mL, 0.05/30 g), animals were placed in a stereotaxic frame (Kopf Instruments) and craniotomy was performed on the right hemisphere. For subicular injections, the following bregma coordinates were used: AP, −3.62 mm; ML, ±2.4 mm; and DV, −1.4 mm or AP, −2.54 mm; ML, ±0.75–0.85 mm; and DV, −1.65 mm. For injections in hippocampal CA1, the coordinates were: AP, −2.44 mm; ML, ± 2.4 mm; and DV, −1.3 mm. The injection pipette, which was attached to a microprocessor-controlled nanoliter injector (Nanoliter 2000; World Precision Instruments), was lowered at a speed of 1 mm/min, and the injection of red IX RetroBeads (Luma Fluor, Inc., a total volume of 25–30 nL) or retrograde viruses (HSV-hEf1-LS1L-GFP, 20 nL; or Cav2-*Cre*; 50 nL) was performed at a rate of 1 nL/s. For both retrograde viruses tested (HSV and Cav2-*Cre*), we detected sparse labelling of local subicular reporter-VIP+ interneurons (Fig. 7b) in addition to CA1 VIP-LRPs. To restrict virus spread, in prior experiments, we estimated the minimal volume of virus required to infect subicular VIP+ cells within a maximum distance of 200 µm from the injection site. The virus spatial labelling efficacy was estimated from the number of subicular cells infected in consecutive coronal sections (50-µm thickness), with ‘zero’ distance corresponding to the injection site (Fig. 7a, left). Ten minutes after the injection, the pipette was slowly withdrawn, the scalp was sutured and the animals were allowed to recover. Two days (for RetroBeads) or 2–3 weeks (for HSV and Cav2-*Cre*) after the injection, the animals were intracardially perfused with 4%-PFA and hippocampal slices were prepared. For all retrograde labelling estimates, only sections from animals with a highly localized subicular injection without spread to the adjoining CA1 area were included in the analysis.

### Electron microscopy

Slices from VIP-eGFP mice containing recorded cells filled with biocytin were re-sectioned to 70 μm, cryoprotected in 20% sucrose solution in 0.1 M PB for ≥3 h and freeze-thawed. Sections were washed in 0.1 M PB and incubated with Streptavidin Alexa 488 (1:1000) in TBS for 48 h at 4 °C. After revealing the biocytin in recorded cells, sections were incubated in biotin (1:100, Vector Labs) overnight followed by the avidin/biotin complex (1:100; Vector Labs) in TBS at 4 °C for 48 h. Sections were reacted with a solution of 0.05% diaminobenzidine and 0.002% hydrogen peroxide (HRP reaction) in Tris buffer for ~10 min After washing in 0.1 M PB, sections were treated with 1% osmium tetroxide solution in 0.1 M PB for 1 h, washed in PB and dehydrated in a graded series of alcohol (70, 90, 95 and 100%) followed by propylene oxide. Uranyl acetate (1%) was added to the 70% alcohol for 35 min for contrast enhancement. Dehydrated sections were embedded in Durcupan resin (Fluka) and polymerized at 60 °C for 2 days. Target areas were cut out from the resin-embedded 70-µm-thick sections and re-embedded for ultramicrotome sectioning. Serial 60-nm-thick sections were cut and mounted on single-slot, pioloform-coated copper grids. Sections were observed with a Philips CM100 transmission electron microscope and electron micrographs were acquired with a Gatan UltraScan 1000 CCD camera. Synaptic junctions were examined in CA1 O/A and RAD. The postsynaptic target identity was determined using published criteria. Briefly, postsynaptic interneuron dendrites receive type 1 (asymmetrical) synapses on the dendritic shafts and show no spines or low spine density. In contrast, postsynaptic PCs receive type 1 synapses on their spines and type 2 symmetrical synapses on their shafts.

### Two-photon imaging in awake mice

Two-photon somatic Ca^2+^-imaging of VIP interneuron activity was performed in head-restrained awake mice running on the treadmill, which consisted of a shock absorber free rotating wheel with minimized brain motion artifacts. The running wheel was equipped with lateral walls for increased animal contentment and coupled with an optical encoder allowing for acquisition of running speed synchronously with electrophysiological signal^39^. Male adult *VIP-Cre* mice (25–35 g body weight; P40–100) were injected stereotaxically with AAV1.Syn.Flex.GCaMP6f.WPRE.SV40 (stock diluted 1:4 in PBS; total injection volume 100 nL) into two sites of the CA1 hippocampus using the following coordinates: AP, −2.54 mm, ML, −2.1 mm, DV, −1.3 mm and AP, −2.0, ML, −1.6, DV, −1.3 mm. At 7–10 days after viral injection, mice were anaesthetized deeply with a ketamine–xylazine mixture (ketamine: 100 mg/kg, xylazine: 10 mg/kg), and fixed in a stereotaxic frame. For hippocampal imaging window, a glass-bottomed cannula was inserted on top of the dorsal hippocampus after the cortex aspiration, and secured with Kwik-Sil at the tissue interface and Superbond at the skull level^39^. For Ca^2+^ imaging from the retrogradely labelled VIP+ O/A interneurons, VIP-*Cre* mice injected with AAV-GCaMP6f in the CA1 were receiving an injection of red RetroBeads (25 nL) in the subiculum (AP, −2.54 mm, ML, −0.85 mm, DV, −1.65 mm) before hippocampal imaging window preparation. A single tungsten electrode for LFP recordings was implanted in the contralateral CA1 hippocampus and a reference electrode was implanted above the cerebellum^39,44^. The head plate was oriented medio-laterally at 7–13° using a four-axis micromanipulator (MX10L, Siskiyou) and fixed with several layers of Superbond and dental cement. Mice were allowed to recover for several days with postoperative pain killer treatment for 3 consecutive days (buprenorphine, 0.1 mg kg^−1^; 48 h). Behavioural habituation involved progressive handling by the experimenter for ~5–15 min twice per day for a total of 3 days, with the animal fixation in the apparatus starting from the 3rd day. During experiment, the LFP signal acquisition was performed simultaneously with the optical encoder signal and imaging trigger at a sampling frequency of 10 kHz using the DigiData 1440 (Molecular Devices), AM Systems amplifier and the AxoScope software (v10.5, Molecular Devices). Imaging was performed using a Leica SP5 TCS two-photon microscope equipped with two external photomultiplier tubes (PMTs) for simultaneous detection of green (GCaMP6f) and red (RetroBeads) fluorescence and coupled with a Ti:sapphire femtosecond laser (Chameleon Ultra II, Coherent), which was mode-locked at 900 nm. A long-range water-immersion 25× objective (0.95 NA, 2.5 mm working distance) was used for excitation and light collection to PMTs at 12 bits. Image series were acquired at axial resolutions of 2 μm/pixel and temporal resolutions of 30–48 images/s. Two 5-min long recording sessions were acquired for each cell. The experiment lasted up to 1 h, after which the mouse was placed back in its home cage. The locomotion wheel between different animals was cleaned with tap water. The image and LFP analyses were performed off-line using Leica LAS, Igor Pro (Wavemetrics, Lake Oswego, USA), Clampfit 10.6 and Statistica (StatSoft).

For post hoc immunohistochemical analysis of VIP-OA interneurons recorded in vivo, a 3D reconstruction of the hippocampal window imaged in vivo was performed using sequential confocal acquisition and automatic stitching. Following in vivo experiments, animals were perfused with 4% PFA, the brains were removed, re-sectioned to 70 µm and processed for GFP, M2R and CR. Sequential confocal Z-stacks (120–150 stacks in total/imaging window, 2-µm step, 500–700-µm depth from the alveus surface) were acquired using Nikon AR1 MP+ multiphoton microscope equipped with a 20× objective (NA 1.1), and automatic stitching of individual Z-stacks was applied using NIS Elements AR 4.51.00 software (Nikon Instruments).

### Analysis of two-photon Ca^2+^ imaging data

For the analysis of spontaneous behaviour, three behavioural phases were identified: locomotion, flickering and immobility. Locomotion epochs were defined as the periods when the instantaneous speed was higher than 2 cm/s for a minimal distance of 2 cm, thereby pooling together the walking and running periods. The periods with small random movements, when the speed was above 0.25 cm/s but below the locomotion threshold, were defined as flickering. Immobility periods were defined as the times without wheel rotation.

The image analysis was performed off-line using Leica LAS, Igor Pro (Wavemetrics, Lake Oswego, USA) and Statistica (StatSoft). Movies were motion corrected along the *x*–*y* plane, no neuropil subtraction was performed^39^. For extraction of somatic Ca^2+^-transients, a region of interest was drawn around individual soma to generate the relative fluorescence change (*F*) vs time trace. The baseline fluorescence level (*F*_0_) was determined as the average fluorescence signal derived from three 1-s time intervals corresponding to the lowest fluorescence level in the absence of Ca^2+^-transients irrespective of the behaviour state. Somatic Ca^2+^-transients were expressed as %Δ*F*/*F* = (*F* − *F*_0_)/*F*_0_ × 100%. Peak Ca^2+^-signals were determined as averaged signals derived from 315-ms windows around the peak of individual Ca^2+^-transients over a total period of locomotion or immobility. A total of 7–10 individual Ca^2+^-transients were analyzed per animal behavioural state with a total of 10–15 states per cell to calculate the average peak Ca^2+^-transient/state for a given cell. This analysis was performed for two independent imaging sessions (S1 and S2, AVG—average between the two; Fig. 5d). Ca^2+^-transient peak amplitudes recorded during two behavioural states (locomotion and immobility) were tested for normality in their distribution using the Shapiro–Wilcoxon test. Otsu’s method based on the discrimination criterion ($\eta = \sigma _B^2/\sigma _T^2$) was applied to somatic Ca^2+^-activity recorded in VIP O/A neurons during locomotion and immobility to identify two types of cells (Fig. 5d). The discrimination criterion between the two groups of cells was 0.86, indicating a good separability. Furthermore, the Mann–Whitney test was used to determine whether obtained groups of neurons exhibit statistically different properties. The results of neuron classification are illustrated as a heat-map with the amplitude of somatic Ca^2+^-fluctuations during different behavioural states colour-coded (Fig. 5d).

To examine somatic Ca^2+^-fluctuations in relation to network oscillations, LFP traces were band-pass filtered to obtain theta oscillations (5–10 Hz) or ripples (125–250 Hz). The frequency of theta oscillations during locomotion or ripple events that were detected during quiet state was determined using the power spectrum analysis in Clampfit. The onset of the theta-run epoch, which was always associated with an increase in theta power, was defined by the beginning of the locomotion period based on the animal speed trace acquired simultaneously with LFP (Fig. 6b, c). Ripple events (9–30 events/cell; minimal event duration: 50 ms; Fig. 6b, c) were selected semi-automatically at 5 SDs above the signal background using Clampfit event search algorithm with a minimal 50-ms spacing interval between individual events. The frequency at the spectral peak of each selected event was confirmed using power spectrum analysis. Ca^2+^-trace segmentation triggered by the event onset (theta-run epoch or ripple) as well as event-triggered averages were conducted in Igor Pro.

### Statistics

The sample size was chosen based on the pilot studies. For statistical analysis, distributions of data were first tested for normality with a Kolmogorov–Smirnov or Shapiro–Wilcoxon test (Figs. 1e, 2e and 5d; Supplementary Tables 1, 3, 4). If data were normally distributed, standard parametric statistics were used: unpaired or paired *t* tests for comparisons of two groups and one-way or repeated-measures ANOVA for comparisons of multiple groups followed by Tukey, Kruskal–Wallis or Chi^2^ tests (Figs. 1e and 2e; Supplementary Tables 1, 3, 4). If data were not normally distributed, non-parametric statistics were used: Mann–Whitney or Wilcoxon’s matched pairs test for comparisons of two groups and Kruskal–Wallis test or Dunn’s test for comparisons of multiple groups (Supplementary Table 1). All statistical analysis was conducted in Sigma Plot 11.0, Igor Pro 4.0 or Statistica. *p*-Values < 0.05 were considered significant. Error bars correspond to SEM.

## Electronic supplementary material

Supplementary Information

## Data Availability

The data sets generated during and/or analyzed during the current study are available from the corresponding author on reasonable request.

## References

[1] Atallah BV, Scanziani M (2009). Instantaneous modulation of gamma oscillation frequency by balancing excitation with inhibition. Neuron.

[2] Lapray D (2012). Behavior-dependent specialization of identified hippocampal interneurons. Nat. Neurosci..

[3] Lovett-Barron M (2012). Regulation of neuronal input transformations by tunable dendritic inhibition. Nat. Neurosci..

[4] Royer S (2012). Control of timing, rate and bursts of hippocampal place cells by dendritic and somatic inhibition. Nat. Neurosci..

[5] Soltesz I (2006). Diversity in the Neuronal Machine: Order and Variability in Interneuronal Microcircuits.

[6] Acsády L, Arabadzisz D, Freund TF (1996). Correlated morphological and neurochemical features identify different subsets of vasoactive intestinal polypeptide-immunoreactive interneurons in rat hippocampus. Neuroscience.

[7] Acsády L, Görcs TJ, Freund TF (1996). Different populations of vasoactive intestinal polypeptide-immunoreactive interneurons are specialized to control pyramidal cells or interneurons in the hippocampus. Neuroscience.

[8] De Marco García NV, Karayannis T, Fishell G (2011). Neuronal activity is required for the development of specific cortical interneuron subtypes. Nature.

[9] Miyoshi G (2015). Prox1 regulates the subtype-specific development of caudal ganglionic eminence-derived GABAergic cortical interneurons. J. Neurosci..

[10] Dávid C, Schleicher A, Zuschratter W, Staiger JF (2007). The innervation of parvalbumin-containing interneurons by VIP-immunopositive interneurons in the primary somatosensory cortex of the adult rat. Eur. J. Neurosci..

[11] Staiger JF, Masanneck C, Schleicher A, Zuschratter W (2004). Calbindin-containing interneurons are a target for VIP-immunoreactive synapses in rat primary somatosensory cortex. J. Comp. Neurol..

[12] Ayzenshtat I, Karnani MM, Jackson J, Yuste R (2016). Cortical control of spatial resolution by VIP+ interneurons. J. Neurosci..

[13] Fu Y (2014). A cortical circuit for gain control by behavioral state. Cell.

[14] Jackson J, Ayzenshtat I, Karnani MM, Yuste R (2016). VIP+ interneurons control neocortical activity across brain states. J. Neurophysiol..

[15] Lee S, Kruglikov I, Huang ZJ, Fishell G, Rudy B (2013). A disinhibitory circuit mediates motor integration in the somatosensory cortex. Nat. Neurosci..

[16] Pfeffer CK, Xue M, He M, Huang ZJ, Scanziani M (2013). Inhibition of inhibition in visual cortex: the logic of connections between molecularly distinct interneurons. Nat. Neurosci..

[17] Pi HJ (2013). Cortical interneurons that specialize in disinhibitory control. Nature.

[18] Bayraktar T, Welker E, Freund TF, Zilles K, Staiger JF (2000). Neurons immunoreactive for vasoactive intestinal polypeptide in the rat primary somatosensory cortex: morphology and spatial relationship to barrel-related columns. J. Comp. Neurol..

[19] Porter JT (1998). Properties of bipolar VIPergic interneurons and their excitation by pyramidal neurons in the rat neocortex. Eur. J. Neurosci..

[20] He M (2016). Strategies and tools for combinatorial targeting of GABAergic neurons in mouse cerebral cortex. Neuron.

[21] Somogyi Peter (2010). Hippocampus: Intrinsic Organization. Handbook of Brain Microcircuits.

[22] Somogyi J (2004). GABAergic basket cells expressing cholecystokinin contain vesicular glutamate transporter type 3 (VGLUT3) in their synaptic terminals in hippocampus and isocortex of the rat. Eur. J. Neurosci..

[23] Karson MA, Tang A, Milner TA, Alger BE (2009). Synaptic cross-talk between perisomatic-targeting interneuron classes expressing cholecystokinin and parvalbumin in hippocampus. J. Neurosci..

[24] Tyan L (2014). Dendritic inhibition provided by interneuron-specific cells controls the firing rate and timing of the hippocampal feedback inhibitory circuitry. J. Neurosci..

[25] Letzkus JJ, Wolff SBE, Lüthi A (2015). Disinhibition, a circuit mechanism for associative learning and memory. Neuron.

[26] Gulyas AI, Hajos N, Katona I, Freund TF (2003). Interneurons are the local targets of hippocampal inhibitory cells which project to the medial septum. Eur. J. Neurosci..

[27] Jinno S (2007). Neuronal diversity in GABAergic long-range projections from the hippocampus. J. Neurosci..

[28] Miyashita T, Rockland KS (2007). GABAergic projections from the hippocampus to the retrosplenial cortex in the rat. Eur. J. Neurosci..

[29] Fuentealba P (2008). Rhythmically active enkephalin-expressing GABAergic cells in the CA1 area of the hippocampus project to the subiculum and preferentially innervate interneurons. J. Neurosci..

[30] Melzer S (2012). Long-range-projecting GABAergic neurons modulate inhibition in hippocampus and entorhinal cortex. Science.

[31] Buzsáki G, Lai-Wo SL, Vanderwolf CH (1983). Cellular bases of hippocampal EEG in the behaving rat. Brain Res. Rev..

[32] Colom LV, Bland BH (1987). State-dependent spike train dynamics of hippocampal formation neurons: evidence for theta-on and theta-off cells. Brain Res..

[33] Ferraguti F (2005). Metabotropic glutamate receptor 8-expressing nerve terminals target subsets of GABAergic neurons in the hippocampus. J. Neurosci..

[34] Cruikshank SJ (2004). Potent block of Cx36 and Cx50 gap junction channels by mefloquine. Proc. Natl. Acad. Sci. U.S.A..

[35] Alvarez VA, Chow CC, Van Bockstaele EJ, Williams JT (2002). Frequency-dependent synchrony in locus ceruleus: role of electrotonic coupling. Proc. Natl. Acad. Sci. U.S.A..

[36] Trenholm S (2014). Nonlinear dendritic integration of electrical and chemical synaptic inputs drives fine-scale correlations. Nat. Neurosci..

[37] Pernelle G, Nicola W, Clopath C (2018). Gap junction plasticity as a mechanism to regulate network-wide oscillations. PLoS Comput. Biol..

[38] Chamberland S, Salesse C, Topolnik D, Topolnik L (2010). Synapse-specific inhibitory control of hippocampal feedback inhibitory circuit. Front. Cell. Neurosci..

[39] Villette V, Levesque M, Miled A, Gosselin B, Topolnik L (2017). Simple platform for chronic imaging of hippocampal activity during spontaneous behaviour in an awake mouse. Sci. Rep..

[40] Chia R, Achilli F, Festing MFW, Fisher EMC (2005). The origins and uses of mouse outbred stocks. Nat. Genet..

[41] Chen XJ (2006). Neuroanatomical differences between mouse strains as shown by high-resolution 3D MRI. NeuroImage.

[42] Mekada K (2009). Genetic differences among C57BL/6 substrains. Exp. Anim..

[43] Otsu N (1979). A threshold selection method from gray-level histogram. IEEE Trans. Syst. Man Cybern..

[44] Malvache A, Reichinnek S, Villette V, Haimerl C, Cossart R (2016). Awake hippocampal reactivations project onto orthogonal neuronal assemblies. Science.

[45] Katona L (2014). Sleep and movement differentiates actions of two types of somatostatin-expressing GABAergic interneuron in rat hippocampus. Neuron.

[46] Varga C, Golshani P, Soltesz I (2012). Frequency-invariant temporal ordering of interneuronal discharges during hippocampal oscillations in awake mice. Proc. Natl. Acad. Sci. U.S.A..

[47] Buzsáki G, Horvath Z, Urioste R, Hetke J, Wise K (1992). High-frequency network oscillation in the hippocampus. Science.

[48] Buzsáki G (2003). Hippocampal network patterns of activity in the mouse. Neuroscience.

[49] Chrobak J, Buzsaki G (1996). High-frequency oscillations in the output networks of the hippocampal-entorhinal axis of the freely behaving rat. Neuroscience.

[50] Villalobos C, Maldonado PE, Valdés JL (2017). Asynchronous ripple oscillations between left and right hippocampi during slow-wave sleep. PLoS One.

[51] Klausberger T, Magill PJ, Cobden PM, Somogyi P (2003). Brain-state- and cell-type-specific firing of hippocampal interneurons in vivo. Nature.

[52] Klausberger T (2004). Spike timing of dendrite-targeting bistratified cells during hippocampal network oscillations in vivo. Nat. Neurosci..

[53] Colom LV, Ford RD, Bland BH (1987). Hippocampal formation neurons code the level of activation of the cholinergic septohippocampal pathway. Brain Res..

[54] Mizumori SJ, Barnes CA, McNaughton BL (1990). Behavioral correlates of theta-on and theta-off cells recorded from hippocampal formation of mature young and aged rats. Exp. Brain Res..

[55] Apostol G, Creutzfeldt OD (1974). Crosscorrelation between the activity of septal units and hippocampal EEG during arousal. Brain Res..

[56] Fukudome Y (2004). Two distinct classes of muscarinic action on hippocampal inhibitory synapses: M2-mediated direct suppression and M1/M3-mediated indirect suppression through endocannabinoid signalling. Eur. J. Neurosci..

[57] Lawrence JJ, Haario H, Stone EF (2015). Presynaptic cholinergic neuromodulation alters the temporal dynamics of short-term depression at parvalbumin-positive basket cell synapses from juvenile CA1 mouse hippocampus. J. Neurophysiol..

[58] Kaifosh P, Lovett-Barron M, Turi GF, Reardon TR, Losonczy A (2013). Septohippocampal GABAergic signaling across multiple modalities in awake mice. Nat. Neurosci..

[59] Unal G, Joshi A, Viney TJ, Kis V, Somogyi P (2015). Synaptic targets of medial septal projections in the hippocampus and extrahippocampal cortices of the mouse. J. Neurosci..

[60] Vervaeke K (2010). Rapid desynchronization of an electrically coupled interneuron network with sparse excitatory synaptic input. Neuron.

[61] Mann-Metzer P, Yarom Y (1999). Electrotonic coupling interacts with intrinsic properties to generate synchronized activity in cerebellar networks of inhibitory interneurons. J. Neurosci..

[62] van Welie I, Roth A, Ho SSN, Komai S, Häusser M (2016). Conditional spike transmission mediated by electrical coupling ensures millisecond precision-correlated activity among interneurons in vivo. Neuron.

[63] Sik A, Penttonen M, Ylinen A, Buzsaki G (1995). Hippocampal CA1 interneurons: an in vivo intracellular labeling study. J. Neurosci..

[64] Ceranik K (1997). A novel type of GABAergic interneuron connecting the input and the output regions of the hippocampus. J. Neurosci..

[65] Deadwyler SA, Hampson RE (2004). Differential but complementary mnemonic functions of the hippocampus and subiculum. Neuron.

[66] Kim SM, Ganguli S, Frank LM (2012). Spatial information outflow from the hippocampal circuit: distributed spatial coding and phase precession in the subiculum. J. Neurosci..

[67] Jackson J, Goutagny R, Williams S (2011). Fast and slow gamma rhythms are intrinsically and independently generated in the subiculum. J. Neurosci..

[68] Jackson J (2014). Reversal of theta rhythm flow through intact hippocampal circuits. Nat. Neurosci..

[69] Taniguchi H (2011). A resource of Cre driver lines for genetic targeting of GABAergic neurons in cerebral cortex. Neuron.

[70] David LS, Topolnik L (2017). Target-specific alterations in the VIP-inhibitory drive to hippocampal GABAergic cells after status epilepticus. Exp. Neurol..

