# Supplementary material for: Connectivity and network state-dependent recruitment of long-range VIP-GABAergic neurons in the mouse hippocampus
Source: Nat Commun. 2018 Nov 28;9:5043. doi: 10.1038/s41467-018-07162-5 (PMC6261953; doi:10.1038/s41467-018-07162-5)
Supplement: Supplementary file 1 — Supplementary Information [file 41467_2018_7162_MOESM1_ESM.pdf]

Supplementary information

Connectivity and network state-dependent recruitment of long-range VIP-GABAergic neurons in the mouse hippocampus

Francavilla R, Villette V. et al.

Contents

Supplementary Figures 1-7

Supplementary Tables 1-4

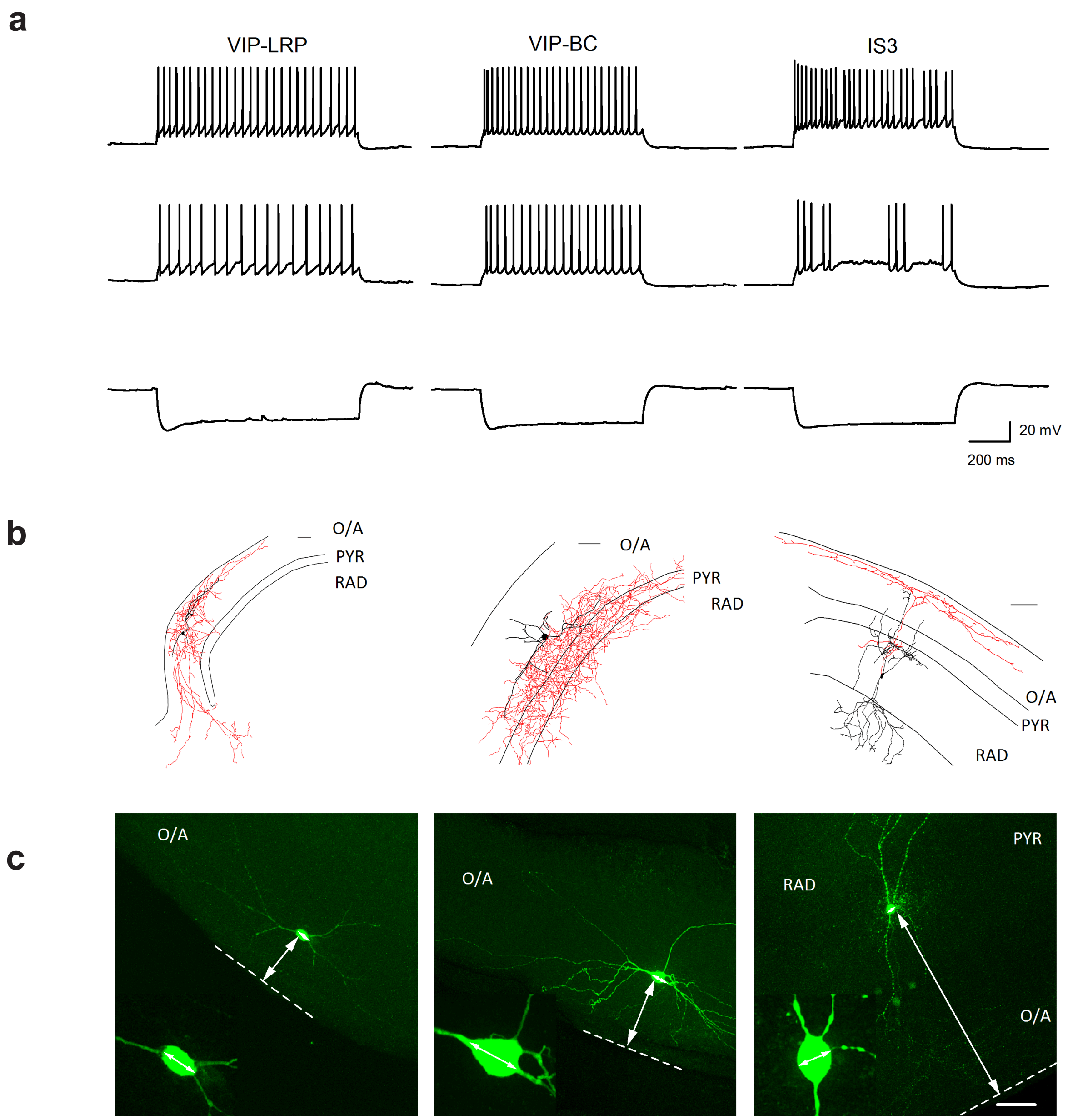

Supplementary Figure 1: Firing properties and anatomy of three types of CA1 VIP+ cells.
(**a**) Example traces of firing properties of VIP-LRP, VIP-BC and IS3 cells. From top to bottom: voltage traces in response to depolarizing and hyperpolarizing current steps injected to the cell soma. (**b**) From left to right: Neurolucida reconstructions of VIP-LRP, VIP-BC and IS3 cells that were recorded and filled with biocytin. Scale bar: 100 µm. (**c**) Confocal images (maximal projection of a Z-stack) illustrating the soma location, size and orientation. The double-arrowhead lines on top of the soma indicate the soma diameter; the double-arrowhead lines between the alveus border and the cell soma illustrate the distance from the soma's location to the alveus. Scale bars: 20 µm for VIP-LRP and VIP-BC and 50 µm for IS3 cell.

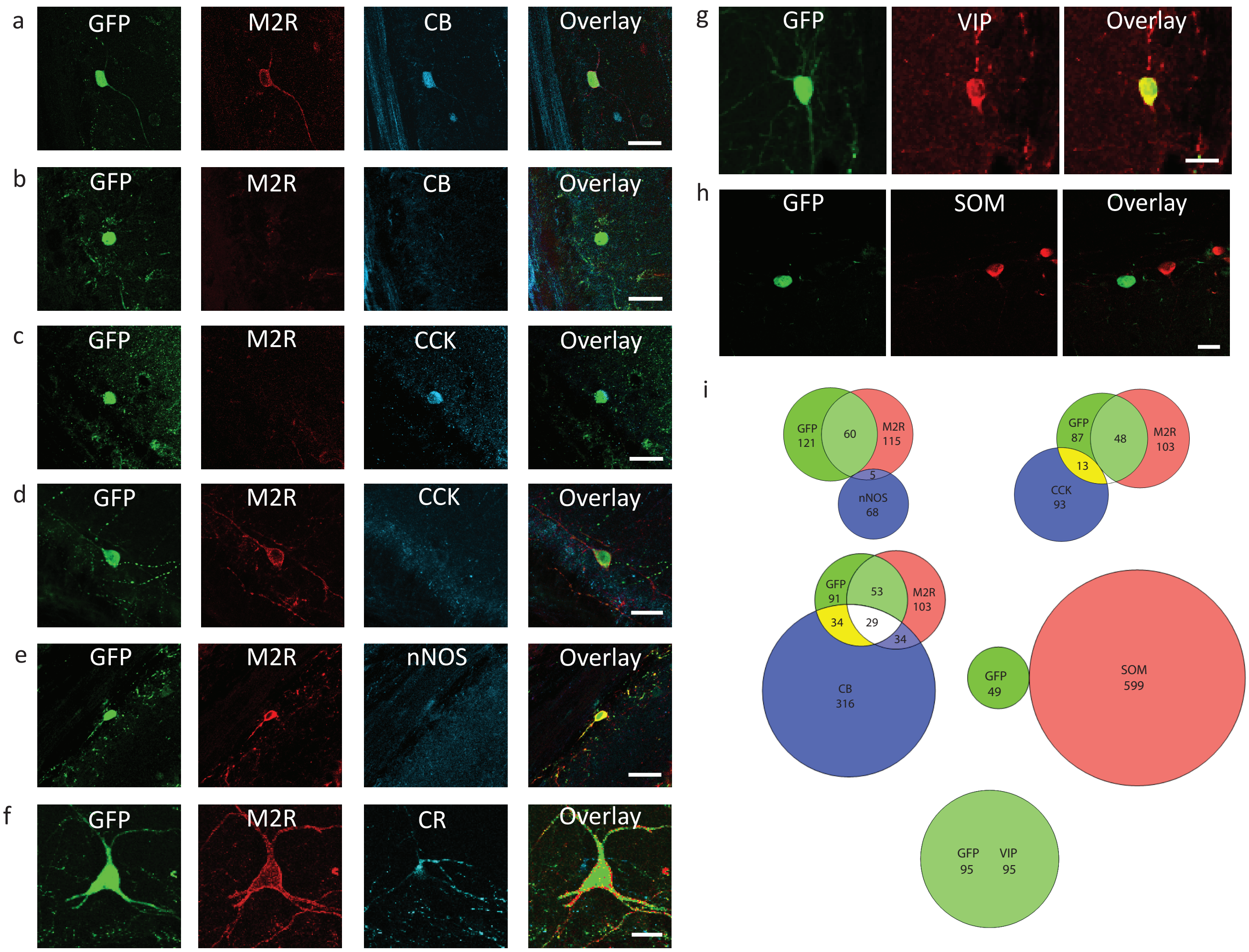

Supplementary Figure 2: Neurochemical properties of VIP+ O/A cells.
(**a-h**) Confocal images (single focal planes) illustrating immunoreactivity for muscarinic acetylcholine receptor 2 (M2R) along with calbindin (CB; panels **a** and **b**), cholecystokinin (CCK; panels **c** and **d**), neuronal nitric oxide synthase (nNOS; panel **e**), calretinin (CR; panel **f**), endogenous VIP (panel **g**) and absence of co-localization for eGFP and somatostatin (SOM; h). Scale bars: scale bar: 50µm (**a-e**), 20 µm (**f-h**). (**i**) Relationships between different markers expressed in VIP+ O/A cells. The total cell number for each marker and the number of co-localized cells are indicated. Markers were tested in separate section sets (20 sections from 3 mice/condition) for each Venn diagram.

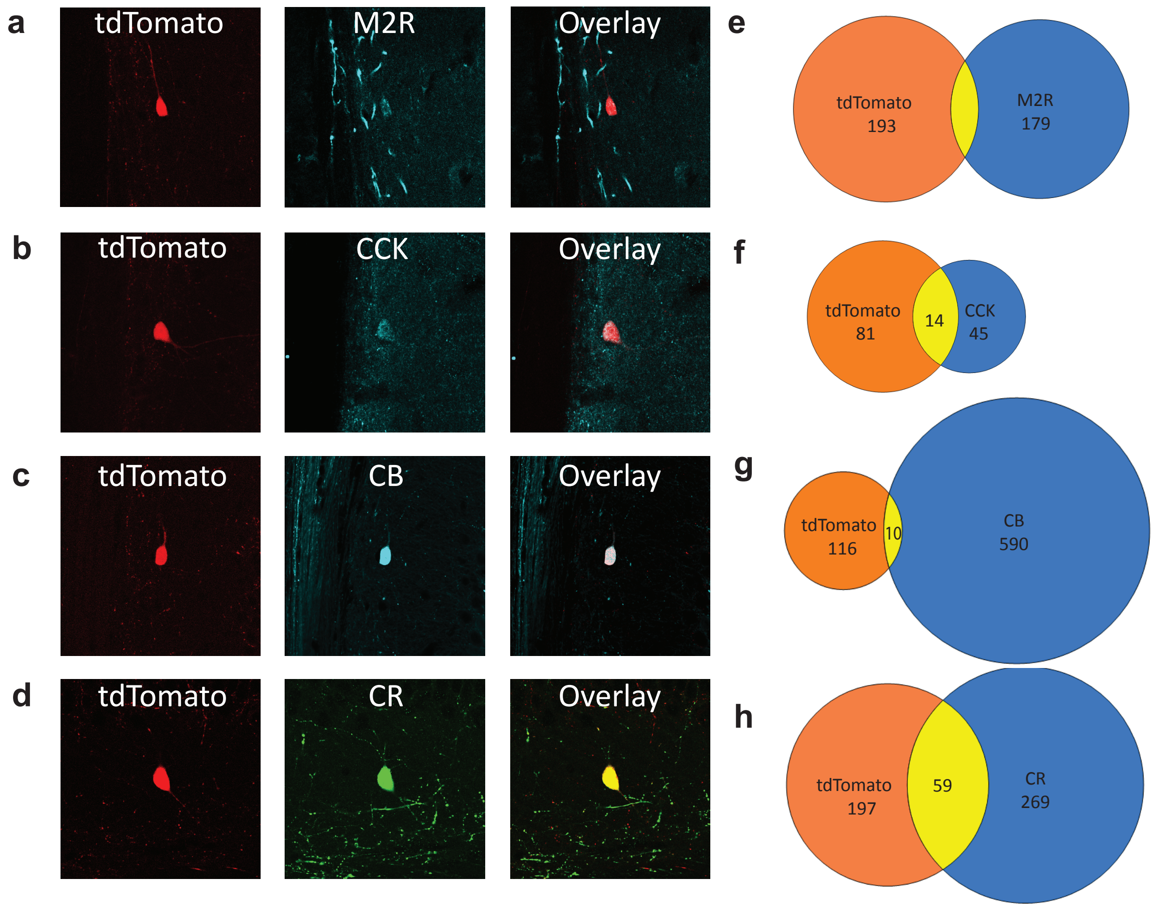

Supplementary Figure 3. Neurochemical properties of VIP+ O/A cells in VIP-Cre;Ai9 mice. (**a-d**) Immunoreactivity for M2R, CCK, CB and CR, scale bar: 20 µm. (**e-h**): Venn diagrams showing the colocalization of different markers in tdTomato+ cells. The total cell number for each marker and the number of co-localized cells are indicated.

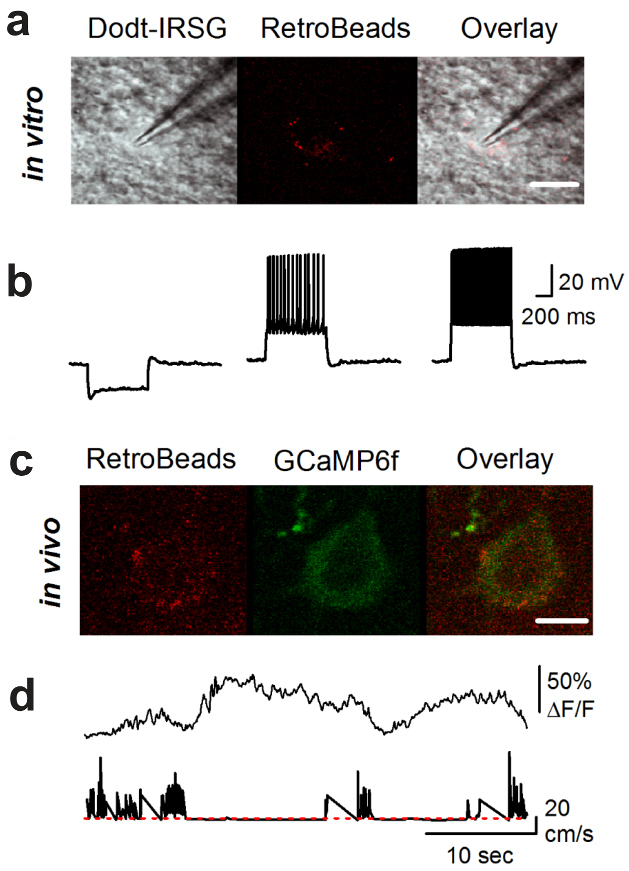

Supplementary Figure 4. Firing properties and activity in vivo of the retrogradely labeled VIP_LRPs

(**a**) Two-photon Dodt-IRSCG and epi-fluorescence images illustrating patch-clamp recording from an interneuron labelled retrogradely with red RetroBeads. (**b**) Sample traces showing the responses to hyperpolarizing (-200 pA) and depolarizing (+200pA, +800 pA) current steps injected to the soma of the cell illustrated in (**a**). (**c,d**) Two-photon in vivo imaging of the retrogradely labelled VIP+ O/A interneurons of VIP-Cre mice with examples of Retro-Bead and GCaMP6f images (**c**) and Ca2+ signals (**d**, upper trace) recorded at different animal speed (**d**, lower trace).

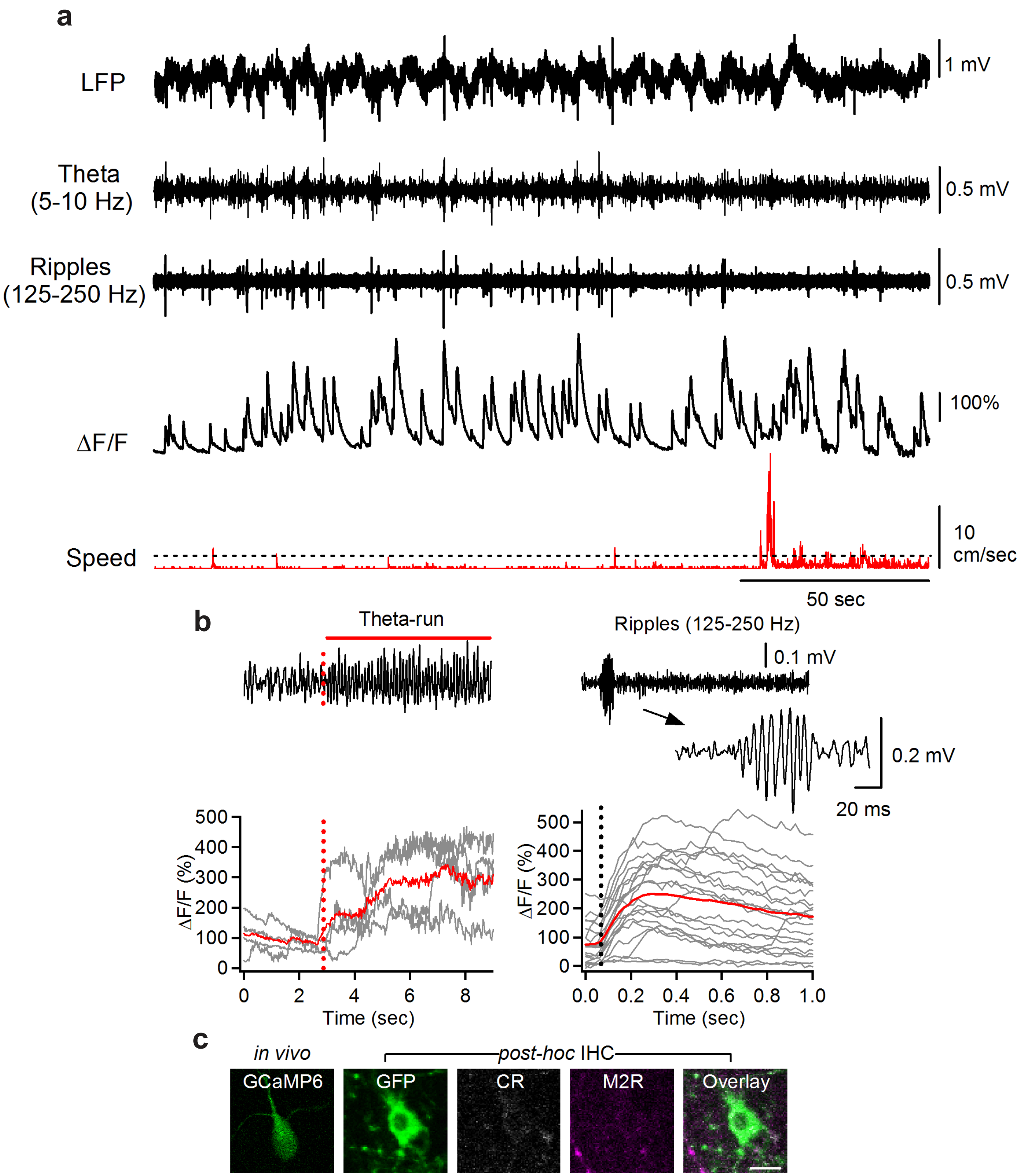

Supplementary Figure 5. Network state-dependent recruitment of an M2R-/CR- VIP interneuron located within PYR.

(**a**) Representative traces of simultaneous LFP (raw trace and filtered for theta and ripples) and Ca2+transient (ΔF/F) recordings from an M2R/CR-negative VIP interneuron (**c**) located in the PYR. Red trace illustrates the animal locomotion speed (dotted line indicates the threshold for locomotion state of 2 cm/sec). (**b**) Individual traces from the event-triggered Ca2+-trace segmentation and corresponding average (red trace) generated by the theta-run epochs (left) and ripples (right) from the cell illustrated in (**a**). (**c**) Posthoc immunohistochemical analysis of the recorded VIP interneuron showing that this cell did not express M2R or CR. Scale bar: 10 µm.

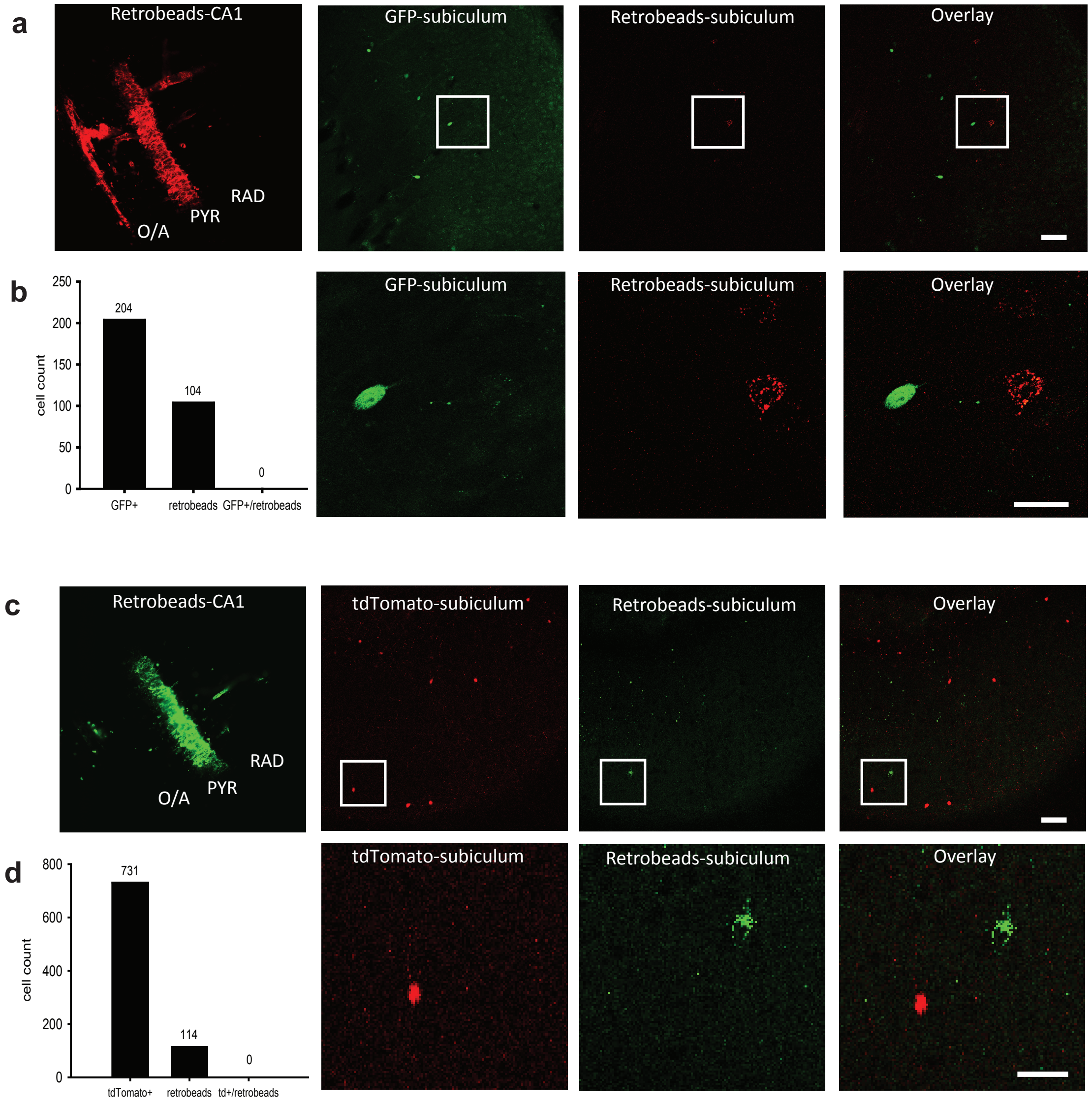

Supplementary Fig. 6. Retrograde labeling in the subiculum following injection in the CA1.
(**a**) Red RetroBeads injection site in the hippocampal CA1 area and the retrograde labelling in the subiculum of VIP-eGFP mice. No VIP-eGFP cell in the subiculum was labelled with RetroBeads, pointing to the absence of CA1-projecting VIP+ cells in the subiculum. (**b**) Summary bar graph showing the total cell number labelled with different markers in the subiculum and the enlarged area from the panel A indicated with white boxes. Scale bar: 100 µm (**a**); 20 µm (**b**). (**c**) Green RetroBeads injection site in the hippocampal CA1 area and the retrograde labelling in the subiculum of VIP-Cre;Ai9 mice. No VIP-tdTomato cell in the subiculum was labelled with RetroBeads, indicating the absence of the CA1-projecting VIP+ cells in the subiculum of this mouse strain. (d) Summary bar graph illustrating the total cell number labelled with different markers in the subiculum and the zoomed area from the panel C indicated with white boxes. Scale bar: 100 µm (**a**); 20 µm (**b**).

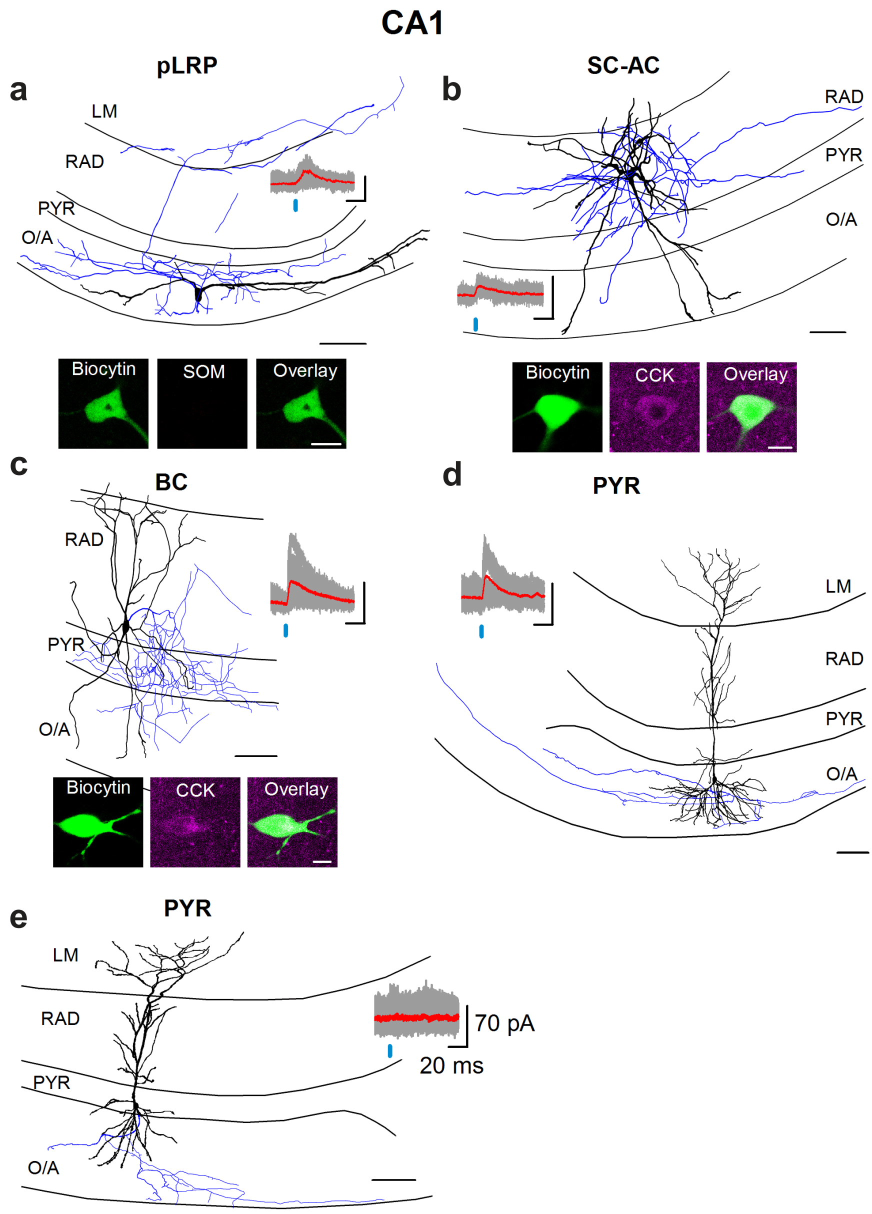

Supplementary Figure 7. Additional VIP-LRP targets revealed using antidromic activation in vitro.
(**a-d**) Examples of the light-evoked IPSCs in response to antidromic activation of VIP-LRPs in different CA1 targets identified anatomically and neurochemically, including a putative LRP neuron (pLRP; **a**), a Schaffer collateral-associated cell (**b**), a CCK-positive BC (**c**) and a pyramidal cell with soma located in stratum oriens (**d**; response was evoked in 2 out of 49 pyramidal cells tested, both were located in stratum oriens). Scale bars: 100 µm. Images at the bottom of each panel show immunoreactivity for SOM (**a**) and CCK (**b, c**). Scale bars: 20 µm (**a**), 10 µm (**b**) and 10 µm (**c**). (**e**) Example illustrating the absence of response in a pyramidal cell with soma in stratum pyramidale. Light-evoked IPSCs were not detected in the majority of CA1 pyramidal cells (47/49 cells).

Supplementary Table 1. Membrane and morphological properties of VIP-LRPs, VIP-BCs and IS3s[a].

	VIP-LRP (n=57)	VIP-BC (n=21)	IS3 (n=19)
**Resting membrane potential (mV)**	-59.5 ± 0.4	-60.5 ± 1.1	-60.7 ± 1.0
**Input resistance (MΩ)**	196.4 ± 10.2	183.9 ± 11.5	537.9 ± 39.4[b] ###^^^
**Membrane capacitance (pF)**	64.7 ± 1.9	58.0 ± 3.1	34.8 ± 1.4 ###^^^
**Action potential threshold (mV)**	-42.7 ± 0.6	-43.0 ± 1.0	-43.1 ± 0.9
**Action potential amplitude (mV)**	65.3 ± 1.0	63.5 ± 2.1	63.9 ± 2.6
**Action potential latency (ms)**	25.9 ± 1.8	24.6 ± 2.3	24.6 ± 3.0
**Action potential half width (ms)**	0.7 ± 0.05	0.7 ± 0.03	0.8 ± 0.3##
**Fast afterhyperpolarization (mV)**	-14.6 ± 0.7	-10.2 ± 0.9[b] ***	-9.9 ± 1.1##
**I_h[c] rectification (mV)**	12.5 ± 0.7	8.8 ± 1.0**	4.1 ± 0.5###^^
**Soma area (µm^2)**	315.0 ±27.4	386.92 ± 36.9	276.08 ± 42.7[d]
**Soma circularity[e]**	0.59 ± 0.04	0.75 ± 0.06	0.73 ± 0.02[d] #
**Soma location (µm)[f]**	59.40 ± 6.0	81.72 ± 11.8	263.30 ± 12.4#^
**Soma orientation (degree)[g]**	77.22 ± 5.4	57.73 ± 9.6	9.75 ± 2.6[d] #^

a. VIP-LRP – vasoactive intestinal peptide (VIP)-expressing long-range projecting cell; VIP-BC – VIP-expressing basket cell; IS3 – type 3 interneuron-selective interneuron (Acsady et al., 1996a).

b. One-way Analysis of Variance test was performed to compare multiple groups. The number of "*" indicates the significance levels of parameters between VIP-LRPs and VIP-BCs, ** – p < 0.01, *** – p < 0.001. The number of "#" indicates the significance levels of parameters between VIP-LRPs and IS3s. # – p < 0.05, ## – p <0.01, ### – p < 0.001. The number of "^" indicates the significance levels of parameters between VIP-BCs and IS3s. ^ – p < 0.05, ^^ – p < 0.01, ^^^ – p < 0.001.

c. Membrane potential 'sag'due to hyperpolarization-activated current measured at -100mV.

d. Normality test failed. Then Dunn's test based on ranking was performed for post-hoc pairwise multiple comparison.

e. The soma circularity was calculated as the ratio between the short and long diameters of the soma.

f. The soma location was determined as the distance from the alveus border.

g. The values of soma orientation indicate the deviation angle from the perpendicular to the alveus edge. See Figure S1C for details.

Supplementary Table 2. Primary and secondary antibodies used.

Primary antibody to	Raised in species	catalog #	RRID	Dilution	Protein conc./quantity original	Source	Reference	Immunogen	Secondary antibodies used
**Calretinin**	goat	sc-11644	AB_634545	1:1000	200 µg/ml	Santa Cruz Biotechnology	Rogers, J.H., 1987	a peptide mapping near the N-terminus of calretinin of human origin	Donkey anti-goat Dylight-650 (Figs. 7D; S2F); Donkey anti-goat Alexa-488 (Fig. S3D)
**Cholecystokinin 26–33 (CCK-8)**	rabbit	C2581	AB_258806	1:800	73 mg/ml	Sigma	Vanderhaeghen et al., 1980	synthetic sulfated cholecystokinin (26-33) amide (sulfated CCK-8), conjugated to KLH	Donkey anti-rabbit Alexa-647 (Figs. S6B-C; S2C-D; S3B)
**Muscarinic 2 receptor**	rat	MAB367	AB_2152546	1:2000	1 mg/mL	Millipore	Levey et al., 1995	i3 loop of m2 receptor fusion protein (225-359), fused to Glutathione S-transferase.	Goat anti-rat Cy3 (Figs. 1D; S2A-E); Donkey anti-rat CF-633 (Figs. 2K; 5G; 6D-E; 7D; S3A; S4C)
**Calbindin**	rabbit	CB 38a	AB_10000340	1:1000	200 µL	Swant	Airaksinen M.S et al., 1997	recombinant rat calbindin D-28k	Donkey anti-rabbit Alexa-647 (Figs. S2A-B; S3C)
**Neuronal nitricoxide synthase**	rabbit	AB5380	AB_91824	1:100	50 µL	Millipore	Kruger, G.M. et al., 2002	Recombinant humanneuronal nitric oxide synthase	Donkey anti-rabbit Alexa-647 (Fig. S2E)
**Parvalbumin**	mouse	P3088	AB_477329	1:1000	27 mg/ml	Sigma	Celio, M. and Heizmann, C., 1981	Purified frog muscle parvalbumin	Donkey anti-mouse Alexa-647 (Fig. 7G)
**Somatostatin**	rat	MAB354	AB_2255365	1:500	100 µL	Millipore	Panula et al., 1986	Synthetic peptide corresponding to amino acids 1-14 of cyclic somatostatin conjugated to bovine thyroglobulin using carbodiimide	Donkey anti-rat Dylight-550 (Figs. 7G-H; S6A); Goat anti-rat Cy3 (Fig. S2H)
**Green fluorescent protein**	chicken	GFP-1020	AB_10000240	1:1000	10 mg/ml	Aves Labs Inc.	Xu et al., 2006	purified recombinant green fluorescent protein (GFP) emulsified in Freund's adjuvant	Donkey anti-chicken Alexa-488 (Figs. 2K; 5G; 6D-E; S2A-H; S4C; S5A)
**Metabotropic glutamate receptor 8**	Goat	sc-30300	AB_2116478	1:1000	200 µg/ml	Santa Cruz Biotechnology	Scherer et al., 1996	a peptide mapping near the C-terminus of GluR-8 of human origin	
**Neuropeptide Y**	rabbit	22940	AB_2307354	1:250	100 µL	ImmunoStar	Jackowski et al., 1989	Neuropeptide Y coupled to bovine thyroglobulin (BTg) with glutaraldehyde	Donkey anti-rabbit Alexa-546 (Fig. 2K)
**Proenkephalin**	rabbit	LS-C23084	AB_902714	1:500	50 µL	LifeSpanBioSciences	Garas et al., 2016	CSYSKEVPEMEKRYGGFMRF conjugated to KLH	Streptavidin-Alexa 546 (Fig. 2K); Streptavidin-Alexa 647 (Fig. 7D)
**Netrin G1**	rabbit	GTX115637	AB_10625511	1:500	0.87 mg/ml	GeneTex	N/A	Recombinant protein encompassing a sequence within the center region of human Netrin G1.	Streptavidin-Alexa 546 (Fig. 2K)
**mGluR1a**	goat	mGluR1a-Go-Af1220	AB_2571800	1:500	200 µg/ml	Frontier Institute	Tanaka et al., 2000	mouse mGluR1a,945-1127 aa (NM_016976)	Donkey anti-goat Cy3 (Fig. 2K)
**mCherry**	rabbit	5993	AB_1975001	1:500	0.5 mg/ml	BioVision	Maus et al., 2015	E. coli expressed recombinant mCherry	Goat anti- rabbit Cy3 (Fig. 7D)
**Vasoactive Intestinal Peptide**	rabbit	20077	AB_572270	1:400	100 µL	ImmunoStar	Buchan et al., 1982	Porcine VIP coupled to bovine thyroglobulin (BTg) with carbodiimide (CDI) linker.	Goat anti- rabbit Cy3 (Fig. S2G)

Supplementary Table 3. Target-specific properties of uIPSCs.

	O-LM (n = 8)	BIS (n = 5)	BC (n = 4)
**Failure rate (%)**	60.1 ± 4.1	44.7 ± 12.9	50.5 ± 9.8
**uIPSCpotency (pA)**	16.3 ± 2.4	32.1 ± 6.2*	26.5 ± 5.9
**Rise time (ms)**	1.0 ± 0.1	0.9 ± 0.1	0.8 ± 0.3
**Decay τ (ms)**	7.8 ± 0.7	9.7 ± 1.2	7.2 ± 1.8
**Latency (ms)**	1.7 ± 0.2	1.6 ± 0.3	1.1 ± 0.1
**PPR (failuresincluded)**	0.9 ± 0.1	0.9 ± 0.03	0.9 ± 0.06

uIPSCs – unitary inhibitory currents, PPR – paired-pulse ratio, O-LM – oriens-lacunosum moleculare cell, BIS – bistratified cell, BC – basket cell; *n* indicates the number of pairs used for the analysis. *$p < 0.05$: OLM *vs* BIS cells.

Supplementary
Table 4. Summary of somatic Ca^{2+} activity for a population of VIP cells recorded within CA1 hippocampal area (CA1, n = 54) and within PYR/RAD (n = 26) vs O/A (n = 28).

	CA1<br><br>mean ± SEM<br>n = 54	CA1 PYR/RAD[a]<br><br>mean ± SEM<br>n = 26	CA1 O/A[a]<br><br>mean ± SEM<br>n = 28
Peak ΔF/F, %<br>Locomotion	88.7 ± 11.8	102.0 ± 22.0	76.4 ± 9.8
Peak ΔF/F, %<br>Immobility	58.0 ± 9.4**[b]	65.2 ± 16.2* [b]	51.3 ± 10.1* [b]

a. PYR/RAD – soma location in stratum pyramidale/ stratum radiatum; O/A – soma location in stratum oriens/ alveus
b. - comparison of peak Ca^{2+} signal (ΔF/F, %) during immobility *vs* locomotion periods within the same cell group (Mann-Whitney test). * -P< 0.05, ** -P< 0.01.
